# Efficient Preparation of α-Ketoacetals

**DOI:** 10.3390/molecules171213864

**Published:** 2012-11-22

**Authors:** Francisco Ayala-Mata, Citlalli Barrera-Mendoza, Hugo A. Jiménez-Vázquez, Elena Vargas-Díaz, L. Gerardo Zepeda

**Affiliations:** Departamento de Química Orgánica, Escuela Nacional de Ciencias Biológicas del IPN, Prol. de Carpio y Plan de Ayala S/N, Col. Santo Tomás, Deleg. Gustavo A. Madero, México, DF 11340, Mexico

**Keywords:** *α*,*α*-dimethoxyacids, Weinreb amide, Grignard reagents, *α*-ketoacetals, salbutamol

## Abstract

The Weinreb amides **2a**,**b** were prepared from the *α,α*-dimethoxyacetic acids **1c**,**d**. A number of representative nucleophilic additions (RMgX and RLi) on **2** afforded *α*-ketoacetals **3a**–**j** in 70–99% yield. These compounds represent a versatile arrangement of functional groups of significant synthetic value, as demonstrated in the synthesis of (±)-salbutamol.

## 1. Introduction

**Scheme 1:**
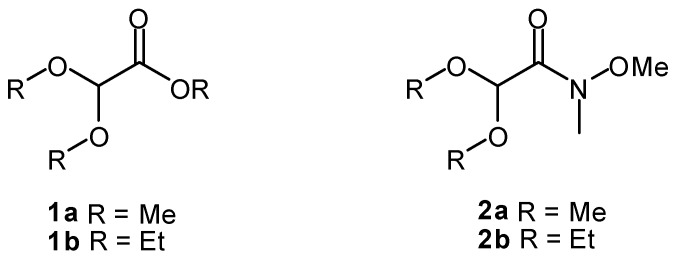
Commercial α,α-dialkoxyacetates **1a**,**b**, available starting materials for the synthesis of Weinreb amides **2a**,**b**.

The *α*-ketoacetals constitute a strategic array of functional groups of great value in synthetic organic chemistry. They offer the possibility of performing the selective functionalization of a keto group over the more reactive aldehyde, as the latter is protected as an acetal. For instance, *α*-ketoacetals are key intermediates in the preparation of chiral cyanohydrins [1], nicotine derivatives [2], chiral sulfoxides [3], α-hydroxy acetals [4,5,6], chiral 1,2-diols [7] and, of particular importance for our research group, of several myrtenal-derived chiral auxiliaries [8,9,10]. A number of methods have been described for the preparation of *α-*ketoacetals, including the classic acetalization of monoalkyl-substituted glyoxals with trialkylorthoformate [1], treatment of *α,α*-dichloroketones with MeONa [11], selenium-catalyzed conversion of terminal alkynes [12] and methyl aryl ketones [13] in the presence of MeOH, transformation of methoxystyrenes with Ce(IV) ammonium nitrate [14], treatment of methylketones with alkylnitrite [15], rearrangement of 1,3-dimethoxy-2-alkanones [16], oxidation of arylketones by thallium(III) and halogens [17], nucleophilic addition to *α,α*-dialkoxyacetyl chlorides [18] as well as addition of RMgX and RLi to ethyl *α,α*-diethoxyacetate [19]. From our own experience, direct treatment of either *α,α*-dialkoxyacetates **1a** or **1b** with Grignard reagents [19] gave the desired α-ketoacetals invariably accompanied by the corresponding tertiary alcohols. Hence, a protocol for the separation of the latter must be implemented in order to obtain pure α*-*ketoacetals. While some methods lack generality because they need specific substrates, others involve elaborate protocols or the formation of byproducts which are hard to separate from the reaction mixture. In addition, there is a scarcity of commercially available α-ketoacetals, which is essentially limited to the existence of 2,2-diethoxyacetophenone and 1,1-dialkoxyacetone [20]. These facts prompted us to develop a general, easy, and efficient procedure to prepare a wide variety of α-keto-acetals. Therefore, we describe herein the preparation of Weinreb amides [21] (WAs) **2a**,**b** (Scheme 1) as key reagents for the synthesis of a wide range of α-ketoacetals through the addition of nucleophiles such as Grignard reagents or alkyllithiums. The synthetic versatility of α-ketoacetals is demonstrated in the synthesis of *rac-*salbutamol. 

## 2. Results and Discussion

**Scheme 2:**
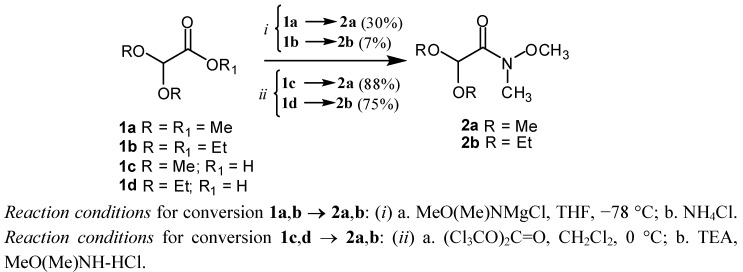
Two explored synthetic routes for preparing Weinreb amides **2a**,**b**.

The preparation of WAs **2a **and **2b** was conceived starting from either commercially available methyl α,α-dimethoxyacetate (**1a**) or ethyl α,α-diethoxyacetate (**1b**) (Scheme 2). Thus, transesterification of **1a** or **1b** with the *N*-magnesium chloride salt of methoxymethyl amine [22,23,24] [formed by treatment of *N,O*-dimethylhydroxylamine (DMHA) with *i*PrMgCl] in anh. THF at −78 °C gave WA **2a** in 30% yield (R = Me), while **2b** (R = Et) was only obtained in trace amounts (*i *pathway, Scheme 2). In order to increase the yield of the WAs **2a** and **2b**, the Ki-Jong [25] protocol was followed, which made use of the also commercially available [26] α,α-diethoxyacetic acids **1c** or **1d** as starting materials. 

Thus, using triphosgene in CH_2_Cl_2_ at 0 °C, followed by the treatment of the carboxylic acid chloride intermediate with MeO(Me)NH-HCl and TEA [25], these compounds were readily converted to their respective WAs **2a** and **2b** in 88 and 75% yield, respectively (*ii* pathway, Scheme 2). The WAs **2a** and **2b** are stable enough to be freely handled without any decomposition under the experimental procedure. Their purification was achieved by distillation using a Kugelrohr apparatus at 40 °C and 0.5 mmHg, or by column chromatography on silica gel.

A representative series of nucleophilic additions performed on WA **2a** yielded only the corresponding α-ketoacetals **3a**–**j**, with yields ranging from good to excellent (Table 1). Both Grignard reagents and alkyllithiums led to essentially the same results, as revealed through trials 1 and 6, and 4 and 8, where these different pairs of nucleophiles gave the same product in very similar yields. The α*-*ketoacetals were obtained in excellent yields and no further purification was required. In the same sense, no substantial differences in the reactivity of WAs **2a** and **2b** were observed, as the latter gave essentially the same result as the former under nucleophilic addition conditions (see entries 1 and 2, 3 and 4, as well as 6 and 7) giving compounds **4a** and **4b**. Although some WAs suffer demethoxylation under the action of LDA or some alkyllithiums (via an E2 reaction) [27], with the concomitant loss of formaldehyde and formation of the corresponding *N*-alkylamide, no such behaviour was observed in this case.

**Scheme 3:**
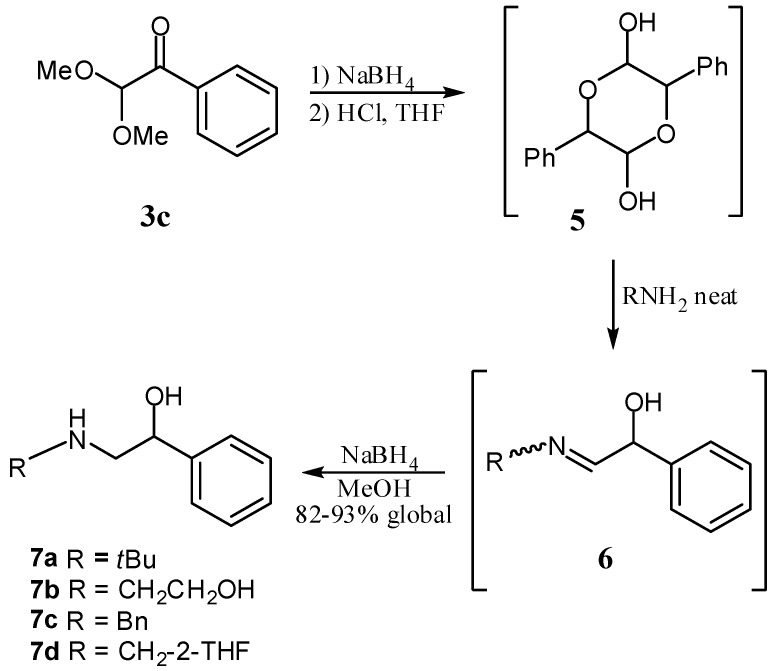
Synthesis of 1,2-aminoalcohols **11a**–**d** from α-ketoacetal **3c**.

In order to illustrate the synthetic utility of α-ketoacetals they were used for the synthesis of 1,2-aminoalcohols in a protocol similar to that previously described (Scheme 3) [28], a method which represents a synthetic alternative to that described by using addition of amines to epoxides [29].

**Scheme 4:**
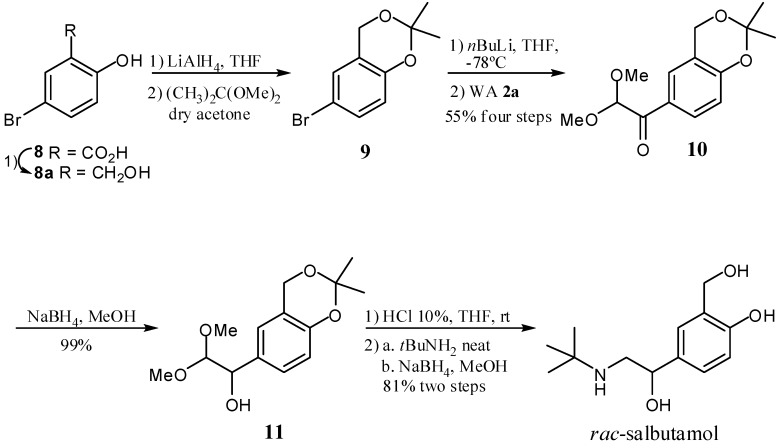
α-Ketoacetal **10** as key intermediate for the synthesis of *rac-*salbutamol.

For instance, α-ketoacetal **3c** was reduced in quantitative yield to the corresponding secondary carbinol, which was hydrolyzed to the cyclic dimer of the corresponding α-hydroxyaldehyde **6**. The latter was directly treated, without isolation, with primary amines followed by reduction of the imine intermediate **6** with NaBH_4_ to afford the corresponding 1,2-aminoalcohols **7a**–**d** in 82–93% yield. This protocol was then successfully implemented for the total synthesis of (±)-salbutamol, a β2-adrenergic receptor agonist used for the treatment of chronic obstructive pulmonary disease. Thus, starting from 6-bromosalicylic acid (**8**), bromobenzodioxane **9** was obtained after reduction of the carboxyl group of **8** and after the successive formation of the dioxane functionality (Scheme 4). Treatment of **9** with Li in dry THF gave the corresponding organolithium which was subsequently added to Weinreb amide **2a** affording the new α-ketoacetal **10** in 55% global yield from salicylic acid **8**. Then, **10** was converted to carbinol **11** with NaBH_4_ in MeOH. Finally, after hydrolysis of **11** and successive treatment of the α-hydroxyaldehyde intermediate with *t*BuNH_2_ and reduction of the corresponding ketoimine with NaBH_4_, (±)-salbutamol was obtained in 81% yield.

**Table 1:** Results of the addition of a representative number of nucleophiles to Weinreb amides **2a**,**b**. 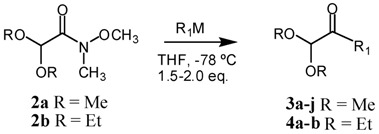

Entry	R_1_M	R_1_	Product (% yield)
1	MeLi	-CH_3_	**3a** (95)
2	MeLi	-CH_3_	**4a** (90) *
3	EtLi	-CH_2_CH_3_	**3b** (99)
4	EtLi	-CH_2_CH_3_	**4b** (89) *
5	PhLi	-C_6_H_5_	**3c** (92)
6	MeMgBr	-CH_3_	**3a** (97)
7	MeMgBr	-CH_3_	**4a** (93)*
8	EtMgBr	-CH_2_CH_3_	**4b** (91)*
9	*n*PrMgBr	-(CH_2_)_2_CH_3_	**3d** (97)
10	CH_3_CCMgBr	-C≡C-CH_3_	**3e** (78)
11	PhCCMgBr	-C≡C-C_6_ H_5_	**3f** (83)
12	4-MeC_6_H_5_MgBr	- *p*C_6_H_4_-CH_3_	**3g** (79)
13	4-FC_6_H_5_MgBr	- *p*C_6_H_4_-F	**3h** (92)
14	3-MeOC_6_H_5_MgBr	- *m*C_6_H_4_-OCH_3_	**3i** (77)
15	BnMgBr	-CH_2_C_6_H_5_	**3j** (81)

* Compounds obtained from **2b** (R = Et).

## 3. Experimental

### 3.1. General Procedures

^1^H and ^13^C-NMR spectra were recorded on Varian spectrometers at 500/125 and 300/75 MHz using CDCl_3_ as solvent and TMS as internal standard. Chemical shift values (δ) are reported in ppm (tetramethylsilane *δ* = 0 ppm for ^1^H; chloroform-d *δ *= 77.0 ppm for ^13^C). Proton spectra are reported as follows: *δ *(multiplicity, number of protons, coupling constant *J*). Multiplicities are indicated by s (singlet), d (doublet), dd (doublet of doublets), t (triplet), q (quartet), st (sextet), m (multiplet), bs (broad signal). Infrared spectra were recorded on a Perkin-Elmer Spectrum 2000 spectrophotometer. High Resolution Mass Spectra (HRMS) were determined with a JEOL GCmate spectrometer by Electronic Impact (EI) ionization. Thin-layer chromatograms (TLC) were done on precoated TLC sheets of silica gel Merck 60F-254. Spots on TLC were revealed by using UV lamp, ceric sulfate, iodine chamber or 2,4-dinitrophenylhydrazine. Purification of compounds was performed by column chromatography on silica gel (Merck 230–400 mesh). A Kugelrohr SEV 200 apparatus was also used for liquid samples. THF was freshly distilled from a purple solution of sodium and benzophenone under nitrogen atmosphere. Some reagents were purchased from Sigma-Aldrich Chemical Co. and used without further purification.

#### N-Trimethoxy-N-methyl-acetamide (**2a**):

##### Method A

*N,O*-Dimethylhydroxylamine hydrochloride (6.70 g, 68.6 mmol) in THF (60 mL) was cooled for 10 min. at −78 °C in a bath of acetone-dry ice. A freshly prepared solution of isopropylmagnesium chloride (14.12g, 137.28 mmol) was slowly added to the above solution, maintaining a vigorous stirring for 30 min under nitrogen atmosphere. To the resulting reaction mixture, methyl dimethoxyacetate (**1a**, 6 mL, 6.58 g, 49.03 mmol) was added dropwise and the reaction mixture was stirred for 1h at −78 °C, then quenched with 20 wt % NH_4_Cl. The reaction was extracted with dichloromethane (3 × 30 mL) and the organic layer was dried over anhydrous Na_2_SO_4_ and evaporated to dryness. The product was purified using a Kugelrohr apparatus at 40 °C (0.5 mmHg), obtaining amide **2a** (2.39 g, 30%) as a pale yellow oil. The same procedure was followed to prepare amide **2b**, starting from ethyl diethoxyacetate (**1b**).

##### Method B

To a stirred solution of the carboxylic acid **1c** (58 mg; 0.48 mmol) in CH_2_Cl_2_ (10 mL) at 0 °C; triphosgene (71.1 mg; 0.24 mmol) and triethylamine (0.334 mL; 2.398 mmol) were added. Then *N,O-*dimethylhydroxylamine hydrochloride (51 mg; 0.52 mmol) was added to the solution and the ice bath removed. The reaction mixture was stirred at room temperature for 1h. The reaction was extracted with EtOAc (3 × 10 mL). Then; the organic phase was dried with anhydrous Na_2_SO_4_ and concentrated *in vacuum*. The product was purified by column chromatography on silica gel using EtOAc-*n-*hexane (1:1) as eluent to give **2a** (69 mg; 88%). R*f* = 0.13 *n-*hexane:EtOAc (4:1). *ν*_max_ (film): 2940; 1679; 1456; 1196; 1066; 977 cm^−1^. ^1^H-NMR (500 MHz; CDCl_3_): *δ* 5.22 (bs; 1H) C-1; 3.75 (s; 3H) MeON; 3.45 (s; 6H) (OCH_3_)_2_; 3.20 (bs; 3H) N-CH_3_. ^13^C-NMR (125 MHz; CDCl_3_): *δ* 167.5 (C-1); 96.3 (C-2); 61.5 (N-OCH_3_); 53.4 ((CH_3_O)_2_); 32.1 (N-CH_3_). EI-HRMS: calculated for C_6_H_13_NO_4_ 163.0845; observed 163.0852.

*2,2-Diethoxy-N-methoxy-N-methyl-acetamide* (**2b**). To a stirred solution of carboxylic acid **1d** (1.27 g, 8.60 mmol) in CH_2_Cl_2_ (35 ml) at 0 °C, triphosgene (1.28 g, 4.3 mmol) and triethylamine (6 mL, 43.0 mmol) were added. Then *N,O-*dimethylhydroxylamine hydrochloride (923 mg, 9.46 mmol) was added to the solution and the ice bath removed. The reaction mixture was stirred at room temperature for 2h, and then filtered, dried with anh. Na_2_SO_4_ and concentrated *in vacuo*. The product was purified by column chromatography on silica gel using EtOAc-*n-*hexane (1:1) to give **2b** (1.24 g, 75%). R*f* = 0.33 *n-*hexane-EtOAc (6:4). *ν*_max_ (film): 2977, 2934, 1683, 1444, 1146, 1062, 987 cm^−1^. ^1^H-NMR (500 MHz; CDCl_3_): *δ* 5.21 (bs, 1H) H-1, 3.61 (s, 3H) NOCH_3_, 3.59 (q, 4H, *J* = 7.0 Hz) 2 OCH_2_, 3.18 (br, 3H) NCH_3_, 1.22 (t, 6H, *J* = 7.0 Hz) (CH_3_)_2_, ^13^C-NMR (125 MHz; CDCl_3_): *δ* 168.0 (C-1), 94.6 (C-2), 63.1 (OCH_2_), 62.2 (NOCH_3_), 33.3 (NCH_3_), 14.8 (CH_3_). EI-HRMS: peak for molecular ion (C_6_H_12_NO_4_) not observed. Calculated for [M−OMe]^+^ 160.0974 (C_7_H_14_NO_3_); observed 160.0974.

### 3.2. General Procedure for the Preparation of α-Ketoacetals

To a solution of amide **2a** (100 mg, 0.61 mmol) in THF (6 mL), cooled at −78 °C, the organometallic reagents (1.5–2.0 eq.) were slowly added, maintaining vigorous stirring under nitrogen atmosphere for 1 h. Then, the reaction was quenched with a saturated solution of NH_4_Cl. The reaction was extracted with dichloromethane (3 × 4 mL). The organic layer was dried over anh. Na_2_SO_4_ and evaporated to dryness. The crude reaction was flash chromatographed (silica gel) using a mixture of *n-*hexane-EtOAc (8:2) as eluent.

*1,1-Dimethoxypropan-2-one* (**3a**). (A) Following the General Procedure described above, *N*-methoxy-*N*-methylacetamide (**2a**, 100 mg, 0.61 mmol) was treated with MeMgBr (0.92 mmol), affording α-ketoacetal **3a** (70 mg, 97%) as a pale yellow liquid. (B) *N*-methoxy-*N*-methylacetamide (**2a**, 100 mg, 0.61 mmol) was treated with MeLi (0.92 mmol), affording α-ketoacetal **3a** (68.7 mg, 95%) as a pale yellow liquid. R*f* = 0.66 *n-*hexane-EtOAc (8:2). ^1^H-NMR (300 MHz; CDCl_3_): *δ* 4.47 (s, 1H) H-1, 3.42 (s, 6H) (OCH_3_)_2_, 2.22 (s, 3H) H-3 [11,30,31,32,33].

*1,1-Dimethoxybutan-2-one* (**3b**). Following the General Procedure described above, *N*-methoxy-*N*-methylacetamide (**2a**, 100 mg, 0.61 mmol) was treated with EtLi (0.92 mmol), affording α-ketoacetal **3b** (80 mg, 99%) as a pale yellow liquid. R*f *= 0.63 *n-*hexane-EtOAc (4:1). ^1^H-NMR (300 MHz; CDCl_3_): *δ* 4.49 (s, 1H) H-1, 3.40 (s, 6H) (OCH_3_)_2_, 2.58 (q, 2H, *J* = 7.5 Hz) H-3, 1.05 (t, 3H, *J* = 7.5 Hz) H-4 [11,30,31,32,33].

*1,1-Diethoxypropan-2-one* (**4a**). (A) Following the General Procedure described above, *N*-methoxy-*N*-methylacetamide (**2b**, 100 mg, 0.52 mmol) was treated with MeMgBr (0.78 mmol), affording α-ketoacetal **4a** (71 mg, 93%). (B) *N*-methoxy-*N*-methylacetamide (**2b**, 100 mg, 0.52 mmol) was treated with MeLi (0.78 mmol), affording α-ketoacetal **4a** (69 mg, 90%). R*f *= 0.33 *n-*hexane-EtOAc (85:15). ^1^H-NMR (500 MHz; CDCl_3_): *δ* 4.25 (s, 1H) H-1, 3.7–3.5 (q, 4H, *J* = 7.0 Hz) (OCH_2_)_2_, 2.18 (s, 3H) H-3, 1.22 (t, 6H, *J* = 7.0 Hz) 2CH_3_ [17,34,35,36].

*1,1-Diethoxybutan-2-one* (**4b**). (A) Following the General Procedure described above, *N*-methoxy-*N*-methylacetamide (**2b**, 100 mg, 0.52 mmol) was treated with EtMgBr (0.78 mmol), affording α-ketoacetal **4b** (75 mg, 90%). (B) *N*-methoxy-*N*-methylacetamide (**2b**, 100 mg, 0.52 mmol) was treated with EtLi (0.78 mmol), affording α-ketoacetal **4b** (74 mg, 89%). R*f *= 0.30 *n-*hexane-EtOAc (85:15). ^1^H-NMR (500 MHz; CDCl_3_): *δ* 4.53 (s, 1H) H-1, 3.45–3.75 (m, 4H) (OCH_2_)_2_, 2.59 (q, 2H, *J *= 7.5 Hz) H-3, 1.20 (t, 6H, *J *= 7.0 Hz) 2CH_3_, 1.01 (t, 3H, *J *= 7.5 Hz) H-4 [11,19,36].

*1,1-Dimethoxy-2-phenylethan-2-one* (**3c**). Following the General Procedure described above, *N*-methoxy-*N*-methylacetamide (**2a**, 100 mg, 0.61 mmol) was treated with PhLi (0.92 mmol), affording α-ketoacetal **3c** (101 mg, 92%) as a colorless liquid. R*f *= 0.51 *n-*hexane-EtOAc (4:1). ^1^H-NMR (500 MHz; CDCl_3_): δ 8.11 (dd, 2H, *J = *7.8, 1.4 Hz) H-*o*, 7.57 (td, 1H, *J *= 7.8, 1.4 Hz) H-*p*, 7.44 (dd, 2H, *J = *7.8, 1.4 Hz) H-*m*, 5.22 (s, 1H) H-1, 3.47 (s, 6H) (OCH_3_)_2_. ^13^C-NMR (125 MHz; CDCl_3_): δ 193.4 (C-2), 133.8 (C-*i*), 133.6 (C-*p*), 129.5 (C-*o*), 128.4 (C-*m*), 103.3 (C-1), 54.5 (OCH_3_)_2_ [1,11,12].

*1,1-Dimethoxypentan-2-one* (**3d**). Following the General Procedure described above, *N*-methoxy-*N*-methylacetamide (**2a**, 100 mg, 0.61 mmol) was treated with *n-*PrMgBr (0.92 mmol), affording α-ketoacetal **3d** (86 mg, 97%) as a pale yellow liquid. R*f *= 0.66 *n-*hexane-EtOAc (4:1). ^1^H-NMR (300 MHz; CDCl_3_): *δ* 4.47 (s, 1H) H-1, 3.41 (s, 6H) (OCH_3_)_2_, 2.54 (t, 2H, *J* = 7.5 Hz) H-3, 1.61 (qui, 2H, *J* = 7.5 Hz) H-4, 0.93 (t, 3H, *J *= 7.5 Hz) H-5. ^13^C-NMR (75 MHz; CDCl_3_): δ 205.7 (C-2), 103.9 (C-1), 54.6 (OCH_3_), 39.2 (C-3), 16.3 (C-4), 13.7 (C-5) [11].

*1,1-Dimethoxypent-3-yn-2-one* (**3e**). Following the General Procedure described above, *N*-methoxy-*N*-methylacetamide (**2a**, 100 mg, 0.61 mmol) was treated with CH_3_CCMgBr (0.92 mmol), affording α-ketoacetal **3e** (67.6 mg, 78%) as a pale yellow liquid. R*f *= 0.40 *n-*hexane-EtOAc (4:1). *ν*_max_ (film): 2931, 2216, 1683, 1455, 1260, 1187, 1118, 1074, 847 cm^−1^. ^1^H-NMR (500 MHz; CDCl_3_): δ 4.60 (s, 1H) H-1, 3.35 (s, 6H) (OCH_3_)_2_, 2.09 (s, 3H) H-5. ^13^C-NMR (125 MHz; CDCl_3_): δ 182.1 (C-2), 103.2 (C-1), 94.7 (C-3), 78.3 (C-4), 54.5 (OCH_3_), 4.5 (C-5). EI-HRMS: calculated for C_7_H_10_O_3_ 142.0630; observed 142.0621.

*1,1-Dimethoxy-4-phenylbut-3-yn-2-one* (**3f**). Following the General Procedure described above, *N*-methoxy-*N*-methylacetamide (**2a**, 100 mg, 0.61 mmol) was treated with PhCCMgBr (0.92 mmol), affording α-ketoacetal **3f** (103.3 mg, 83%) as a pale yellow solid. R*f* = 0.40 *n*-hexane-EtOAc (4:1). *ν*_max_ (film): 2918, 2204, 1679, 1489, 1444, 1070, 758, 689 cm^−1^. ^1^H-NMR (500 MHz; CDCl_3_): δ 7.27–7.43 (m, 5H) Ar, 4.76 (s, 1H) H-1, 3.50 (s, 6H) (OCH_3_)_2_. ^13^C-NMR (125 MHz; CDCl_3_): *δ* 182.3 (C-2), 133.4 (C-*o*), 131.1 (C-*p*), 128.6 (C-*m*), 119.6 (C-*i*), 103.00 (C-1), 94.9 (C-4), 86.4 (C-3), 54.4 (OCH_3_)_2_. EI-HRMS: calculated for [M-OMe]^+·^ (C_11_H_9_O_3_) 173.0603; observed 173.0607.

*1,1-Dimethoxy-2-(4-methylphenyl)-ethan-2-one* (**3g**). Following the General Procedure described above, *N*-methoxy-*N*-methylacetamide (**2a**, 100 mg, 0.61 mmol) was treated with 4-Me-C_6_H_4_MgBr (0.92 mmol), affording α-ketoacetal **3g** (94 mg, 79%) as a pale yellow liquid. R*f* = 0.46 *n*-hexane-EtOAc (4:1). ^1^H-NMR (500 MHz; CDCl_3_): δ 8.12 (d, 2H, *J = *8.1 Hz) H-*o*, 7.36 (d, 2H, *J = *8.1 Hz) H-*m*, 5.23 (s, 1H) H-1, 3.48 (s, 6H) (OCH_3_)_2_, 2.43 (s, 3H) CH_3_. ^13^C-NMR (125 MHz; CDCl_3_): δ 193.0 (C-2), 144.6 (C-*p*), 131.3 (C-*i*), 129.6 (C-*o*), 129.2 (C-*m*), 103.1 (C-1), 54.4 (OCH_3_)_2_, 21.7 (CH_3_) [37,38,39].

*1,1-Dimethoxy-2-(4-fluorophenyl)-ethan-2-one* (**3h**). Following the General Procedure described above, *N*-methoxy-*N*-methylacetamide (**2a**, 100 mg, 0.61 mmol) was treated with 4-F-C_6_H_4_MgCl (0.92 mmol), affording α-ketoacetal **3h** (112 mg, 92%) as a pale yellow liquid. R*f* = 0.44 *n*-hexane-EtOAc (4:1). ^1^H-NMR (500 MHz; CDCl_3_): 8.17 (m, 2H) H-*o*, 7.13 (m, 2H) H-*m*, 5.12 (s, 1H) H-1, 3.48 (s, 6H) (OCH_3_)_2_. ^13^C-NMR (125 MHz; CDCl_3_): δ 191.9, (C-2), 167.0 (*^1^J* (C-F) = 254.3 Hz, C-*p*), 132.4 (*^3^J* (C-F) = 9.3 Hz, C-*o*), 130.1 (*^4^J* (C-F) = 3 Hz, C-*i*), 115.5 (*^2^J* (C-F) = 21.6 Hz, C-*p*), 104.1 (C-1), 54.1 (OCH_3_)_2_ [37,38,39].

*1,1-Dimethoxy-2-(3-methoxyphenyl)ethan-2-one* (**3i**). Following the General Procedure described above, *N*-methoxy-*N*-methylacetamide (**2a**, 100 mg, 0.61 mmol) was treated with 3-MeO-C_6_H_4_MgBr (0.92 mmol), affording α-ketoacetal **3i** (99 mg, 77%) as a pale yellow liquid. R*f* = 0.37 *n*-hexane-EtOAc (4:1). ^1^H-NMR (500 MHz; CDCl_3_): δ 7.72 (dd, 1H, *J = *8.0, 1.5 Hz) H-6', 7.61 (dd, 1H, *J = *2.7, 1.5 Hz) H-2', 7.37 (t, 1H, *J = *8.0 Hz) H-5', 7.13 (dd, 1H, *J = *8.0, 2.7 Hz) H-4', 5.23 (s, 1H) H-1, 3.86 (s, 3H) ArOCH_3_, 3.47 (s, 6H) (OCH_3_)_2_. ^13^C-NMR (125 MHz; CDCl_3_): δ 193.2 (C-2), 159.6 (C-3'), 135.0 (C-1'), 129.4 (C-5'), 122.2 (C-6'), 120.3 (C-4’), 113.5 (C-2'), 103.0 (C-1), 55.3 (-C_6_H_4_-OCH_3_), 54.4 (OCH_3_)_2_ [40,41].

*1,1-Dimethoxy-3-phenilpropan-2-one* (**3j**). Following the General Procedure described above, *N*-methoxy-*N*-methylacetamide (**2a**, 100 mg, 0.61 mmol) was treated with BnMgCl (0.92 mmol), affording α-ketoacetal **3j** (96 mg, 81%) as a pale yellow liquid. R*f* = 0.44 *n*-hexane-EtOAc (4:1). ^1^H-NMR (500 MHz; CDCl_3_): *δ *7.15–7.35 (m, 5H) Ar, 4.53 (s, 1H) H-1, 3.86 (s, 2H) H-3, 3.41 (s, 6H) (OCH_3_)_2_. ^13^C-NMR (125 MHz; CDCl_3_): *δ* 202.6 (C-2), 133.4 (C-*i*), 129.7 (C-*m*), 128.5 (C-*o*), 126.9 (C-*p*), 103.6 (C-1), 54.7 (OCH_3_)_2_, 44.1 (C-3) [12,17,42].

### 3.3. General Procedure for the Synthesis of β-Aminoalcohols

To a cooled (0 °C) solution of α,α-dimethoxyacetophenone (**3c**, 2 g, 11.09 mmol) in EtOH (100 mL) 839.3 mg (22.18 mmol) of NaBH_4_ were added and the resulting mixture was stirred for 30 min. The reaction was quenched with acetone, the solvent was evaporated and 100 mL of hot water was added to the crude reaction. The latter was extracted with dichloromethane (3 × 5 mL), the organic layer was dried over anh. Na_2_SO_4_ and evaporated to dryness giving the corresponding carbinol (1.98 g, >96%). This compound (500 mg, 2.74 mmol) was treated with 0.2 mL of HCl 37% diluted in 2 mL of THF and stirred for 15 min. The reaction mixture was washed with a sat. solution of NaHCO_3_ (3 × 10 mL), extracted with dichloromethane (3 × 15 mL) and the organic layer was dried over anh. Na_2_SO_4_ and evaporated to dryness, giving 285.9 mg (74%) of **5** as a white solid.

The amine (7.34 mmol) was added to a solution of α-hydroxyaldehyde **5 **(200 mg, 1.49 mmol) in THF and the resulting mixture was stirred for 30 min. The crude reaction was dissolved in 3 mL of ethanol and treated with NaBH_4_ (2.94 mmol) under vigorous stirring for 30 min. After this time, the reaction was quenched with acetone and the solvent was evaporated. The crude syrup was treated with 5 mL of hot water, extracted with dichloromethane and (3 × 5 mL), the organic layer was dried over anh. Na_2_SO_4_ and evaporated to dryness giving the corresponding β-aminoalcohol.

*2-(tert-Butylamino)-1-pheny-1-phenylethanol* (**7a**). Following the General Procedure as described above, intermediate **5** (200 mg, 1.49 mmol) was treated with *t-*BuNH_2_ (7.3 mmol). The product was purified by column chromatography on silica gel using ethanol-dichloromethane (3:7) to give **7a** (254 mg, 90%) as a white solid. ^1^H-NMR (300 MHz; CDCl_3_): δ 7.38 (m, 5H) Ar-H, 4.62 (dd, 1H, *J *= 8.8, 3.7 Hz) H-1, 2.90 (dd, 1H, *J = *12.0, 3.7 Hz) H-2a, 2.89 (s, 1H) OH, 2.60 (dd, 1H, *J* =12.0, 8.8 Hz) H-2b, 1.10 (s, 9H) *t*-Bu [18,43,44,45].

*2-(2-Hydroxyethyl)-1-phenylethanol* (**7b**). Following the General Procedure described above, α-hydroxyaldehyde **5 **(200 mg, 1.49 mmol) was treated with ethanolamine (7.3 mmol). The product was recrystallized using *n*-hexane, giving **7b** (218 mg, 82%). ^1^H-NMR (300 MHz; CDCl_3_): δ 7.35 (m, 5H) Ar-H, 4.76 (dd, 1H, *J *= 8.8, 3.7 Hz) H-1, 3.69 (t, 2H, *J* = 5.1 Hz) H-3, 2.90 (m, 4H) H-2 and H-4, 2.30 (bs, 3H) NH, 2OH [46,47].

*2-(Benzylamino)-1-phenylethanol* (**7c**). Following the General Procedure described above, α-hydroxyaldehyde **5** (200 mg, 1.49 mmol) was treated with benzylamine (7.3 mmol). The product was recrystallized using *n*-hexane and a small amount of dichloromethane, giving **7c** (311 mg, 93%) as a white solid. ^1^H-NMR (300 MHz; CDCl_3_): δ 7.5–7.10 (m, 10H) Ar-H, 4.73 (dd, 1H, *J *= 8.9, 3.6 Hz) H-1, 3.85 (m, 2H) H-3, 2.94 (dd, 2H, *J *= 12.2, 3.6 Hz) H-2a, (dd, 2H, *J *= 12.2, 8.9 Hz) H-2b, 2.24 (bs, 2H) OH, NH [48,49,50,51].

*1-Phenyl-2-((tetrahydrofuran-2-yl)methylamino) ethanol* (**7d**). Following the General Procedure described above, α-hydroxyaldehyde **5** (200 mg, 1.49 mmol) was treated with 2-tetrahydrofurfurylamine (7.3 mmol). The product was recrystallized using *n*-hexane, giving **7d** (283 mg, 87%) as a white solid. ^1^H-NMR (300 MHz; CDCl_3_): δ 7.35 (m, 5H) Ar-H, 4.70 (dd, 1H) H-1, 4.01 (m, 1H) H-4, 3.79 (m, 2H) H-7a,b, 2.92 (m, 1H) H-2a, 2.71 (m, 3H) H-3a,b and H-2b, 2.40 (br, 2H) NH and OH, 2.00–1.42 (m, 4H) H-5a,b and H-6a,b [52].

*Bromo-2-(hydroxymethyl)phenol* (**8a**). A solution of LiAlH_4_ (3.5 g, 90 mmol) in 50 mL of ether was cooled for 30 min. at −78 °C in a bath of acetone-dry ice. After that, a solution of 5-bromosalicylic acid (**8**, 16 g, 0.078 mmol) in ether (20 mL) was added dropwise and the reaction mixture was stirred for 2.5 h under nitrogen atmosphere and was quenched with EtOAc and water (ice). Then, to the reaction mixture a solution of hydrochloric acid 50% v/v (200 mL) was added. The reaction was extracted with dichloromethane (3 × 20 mL) and washed with sat. NaHCO_3_ (3 × 30 mL) and the organic layer was dried over anh. Na_2_SO_4_ and evaporated to dryness. The crude was dissolved in EtOAc and *n*-hexane was added to obtain a precipitate, obtaining **8a** (8.6 g, 57%) as a white solid. ^1^H-NMR (500 MHz; CDCl_3_): δ 9.80 (br, 1H) OH, 7.31 (d, 1H, *J *= 2.5 Hz) H-3, 7.20 (dd, 1H, *J* = 8.6, 2.5 Hz) H-5, 6.82 (d, 1H, *J* = 8.6 Hz) H-6, 5.08 (br, 1H) OH, 4.42 (s, 2H) CH_2_ [53,54,55].

*6-Bromo-2,2-dimethyl-4H-benzo[d][1,3]dioxane* (**9**). To a stirred solution of **8a** (5 g, 24.62 mmol), *p*-TsOH (450 mg, 0.24 mmol) and sodium sulfate (9.6 g) in acetone (95 mL) 2,2-dimethoxypropane (121 mmol) were added. The reaction was maintained with a vigorous stirring for 72 h at 40 °C. After that, the reaction was extracted with CH_2_Cl_2_ (120 mL) and washed with sat. NaHCO_3_ (3 × 30 mL). The organic layer was dried over anh. Na_2_SO_4_ and evaporated to dryness giving **9** (5.65 g, quantitative yield) as an amber liquid. ^1^H-NMR (500 MHz; CDCl_3_): δ 7.21 (dd, 1H, *J *= 8.5, 2.5 Hz) Hb, 7.03 (d, 1H, *J* = 2.5 Hz) Hc, 6.70 (d, 1H, *J* = 8.6 Hz) Ha, 4.78 (s, 2H) CH_2_, 1.51 (s, 6H) 2CH_3_ [56].

*1-(2,2-Dimethyl-4H-benzo[d][1,3]dioxin-6-yl)-2,2-methoxyethanone* (**10**). To a solution of **9** (163 mg, 67 mmol) in THF (7 mL) at −78 °C, 1.6 M *n*BuLi (0.92 mL 1.47 mmol) was slowly added maintaining a vigorous stirring under nitrogen atmosphere for 45 min. This solution was added dropwise to a solution of **2a** (109 mg, 0.67 mmol) in 10 mL of THF cooled at −78 °C. The reaction was stirred under nitrogen atmosphere for 1 h. Then the reaction was quenched with a saturated solution of NH_4_Cl. The reaction was extracted with dichloromethane (3 × 5 mL), the organic layer was dried over anh. Na_2_SO_4_ and evaporated to dryness giving **10** (176 mg, quantitative yield) as a yellow syrup. *ν*_max_ (film): 1693, 1497, 1375, 1272, 1204, 1110, 1067, 955, 433 cm^−1^. ^1^H-NMR (300 MHz; CDCl_3_): δ 7.98 (dd, 1H, *J *= 7.5, 2.3 Hz) H-2', 7.83 (d, 1H, *J *= 2.3 Hz) H-6', 6.85 (d, 1H, *J *= 7.5 Hz) H-3', 5.15 (s, 1H) H-1, 4.88 (s, 2H) H-7, 3.46 (s, 6H) (OCH_3_)_2_, 1.56 (s, 6H) 2CH_3_. ^13^C-NMR (75 MHz; CDCl_3_): *δ* 191.9 (C-2), 156 (C-1'), 130.2 (C-5'), 127.1 (C-6'), 126.1 (C-4'), 118.9 (C-2'), 117.0 (C-3'), 103.6 (C-1), 100.5 (C-8'), 60.6 (C-7'), 54.5 ((OCH_3_)_2_), 24.7 (2 CH_3_). EI-HRMS: calculated for C_14_H_18_O_5_ 266.1154; observed 266.1154.

*1-(2,2-Dimethyl-4H-benzo[d][1,3]dioxin-6-yl)-2,2-dimethoxyethanol* (**11**). To a cooled solution (0 °C) of **10** (161 mg, 0.6 mmol) in ethanol (10 mL) NaBH_4_ (46 mg, 1.22 mmol) was added. The reaction mixture was stirred for 30 min. Then it was quenched with acetone, the solvent was evaporated and 5 mL of hot water was added to the reaction crude. The reaction was extracted with dichloromethane (3 × 5 mL), the organic layer was dried over anh. Na_2_SO_4_ and evaporated to dryness giving **11** (160 mg, quantitative yield) as a yellow and viscous liquid. ^1^H-NMR (300 MHz; CDCl_3_): δ 7.18 (dd, 1H, *J *= 8.4, 1.6 Hz) H-2', 7.02 (d, 1H, *J *= 1.6 Hz) H-6', 6.78 (d, 1H, *J *= 8.4 Hz) H-3', 4.83 (s, 2H) H-7', 4.50 (d, 1H, *J *= 6.5 Hz) H-2, 4.24 (d, 1H, *J *= 6.5 Hz) H-1, 3.45 (s, 3H) OCH_3_, 3.25 (s, 3H) OCH_3_, 2.90 (br, 1H) OH, 1.52 (s, 6H) 2CH_3_. ^13^C-NMR (75 MHz; CDCl_3_): *δ* 150.7 (C-4'), 131.2 (C-1'), 126.9 (C-2'), 123.2 (C-6'), 118.9 (C-5'), 116.6 (C-3'), 107.5 (C-1), 99.3 (C-8'), 73.3 (C-2), 60.7 (C-7'), 55.7 (OCH_3_), 54.7 ((OCH_3_)_2_), 24.7 (2CH_3_), 24.4 (CH_3_). EI-HRMS: calculated for C_14_H_18_O_5_ 268.1311; observed 268.1311.

(±)-*Salbutamol*. To a solution of hydroxyacetal **11** (160 mg, 0.59 mmol) 37% HCl (0.16 mL) diluted in THF (1 mL) was added. After 15 min the reaction finished and the reaction mixture was washed with a solution of NaHCO_3_ sat (3 × 2 mL). The reaction was extracted with dichloromethane and (3 × 3 mL) the organic layer was dried over anh. Na_2_SO_4_ and evaporated to dryness, giving the corresponding α-hydroxyaldehyde (80 mg as crude). To this crude *t-*BuNH_2_ (0.76 mL, 7.3 mmol) was added and the reaction mixture was stirred for 30 min. The reaction crude was dissolved in ethanol (3 mL) and treated with NaBH_4_ (109.7 mg, 2.9 mmol) under vigorous stirring for 30 min. The reaction was then quenched with acetone and the solvent was evaporated. After this, hot water (5 mL) was added to the crude. The reaction was extracted with dichloromethane and (3 × 5 mL). The organic layer was dried over anh. Na_2_SO_4_ and evaporated to dryness giving (*rac)-*salbutamol (85.3 mg, 81% yield). ^1^H-NMR (300 MHz; CDCl_3_): δ 7.11 (dd, 1H, *J* = 8.1, 1.8 Hz) H-2', 7.03 (d, 1H, *J *= 1.8 Hz) H-6', 6.8 (dd, 1H, *J *= 8.1Hz) H-3', 4.53 (dd, 1H, *J *= 8.8, 3.7 Hz) CHOH, 3.90 (s, 2H) CH_2_OH, 2.90 (dd, 1H, *J *= 12.0, 3.7 Hz) NHCH_2_, 2.60 (dd, 1H, *J *= 12.0, 8.8 Hz) NHCH_2_, 2.2 (br, 4H) NH and 3(OH), 1.10 (s, 9H) *t*-Bu [57].

## 4. Conclusions

In conclusion, it has been shown that WAs **2a,b** represent an efficient and practical alternative for obtaining a wide variety of α*-*ketoacetals, which in turn represent an array of functional groups in high demand in synthetic organic chemistry. A practical synthetic application of α-ketoacetals was developed for the synthesis of some 1,2-aminoalcohols, including the total synthesis of (±)-salbutamol.

## Supplementary Materials

Supplementary materials can be accessed at: http://www.mdpi.com/1420-3049/17/12/13864/s1.

## References

[1] Qin B., Liu X., Shi J., Zheng K., Zhao H., Feng X. (2007). Enantioselective Cyanosilylation of α,α-Dialkoxy Ketones Catalyzed by Proline-Derived in-Situ-Prepared *N*-Oxide as Bifunctional Organocatalyst. J. Org. Chem..

[2] Graef E., Troschuetz R. (1999). Synthesis of 6-Phenyl Substituted 2-Formylnicotinates. Synthesis.

[3] Garcia Ruano J.L., Maestro M.C., Sanchez-Sancho F. (1995). Enantiomerically pure 3-p-tolylsulfinyl acrolein and crotonaldehyde dimethylacetals. Stereoselective reduction of β-keto-γ,γ-dialkoxysulfoxides. Tetrahedron: Asymmetry.

[4] Török B., Balázsik K., Bartók M., Felföldi K., Bartók M. (1999). New synthesis of a useful C3 chiral building block by a heterogeneous method: enantioselective hydrogenation of pyruvaldehyde dimethyl acetal over cinchona modified Pt/Al2O3 catalysts. Chem. Commun..

[5] Studer M., Burkhardt S., Blaser H.-U. (1999). Enantioselective hydrogenation of alfa-keto acetals with cinchona modified Pt catalyst. Chem. Commun..

[6] Szőllősi G., Makra Z., Fülöp F., Bartók M. (2011). The First Case of Competitive Heterogeneously Catalyzed Hydrogenation using Continuous-Flow Fixed-Bed Reactor System: Hydrogenation of Binary Mixtures of Activated Ketones on Pt-Alumina and on Pt-Alumina-Cinchonidine Catalysts. Catal. Lett..

[7] Tamura Y., Kondo H., Annoura H., Takeuchi R., Fujioka F. (1986). Diastereoselective nucleophilic addition to chiral open-chain α-ketoacetals: Synthesis of (*R*)- and (*S*)-mevalolactone. Tetrahedron Lett..

[8] Becerra-Martínez E., Velázquez-Ponce P., Sánchez-Aguilar M.A., Rodríguez-Hosteguín A., Joseph-Nathan P., Tamariz J., Zepeda L.G. (2007). New 2-acyl-1,3-dioxane derivatives from (1R)-(−)-myrtenal: Stereochemical effect on their relative ability as chiral auxiliaries. Tetrahedron: Asymmetry.

[9] Vargas-Díaz M.E., Joseph-Nathan P., Tamariz J., Zepeda L.G. (2007). Synthesis of a New (1*R*)-(−)-Myrtenal-Derived Dioxadithiadodecacycle and Its Use as an Efficient Chiral Auxiliary. Org. Lett..

[10] Vargas-Díaz M.E., Lagunas-Rivera S., Joseph-Nathan P., Tamariz J., Zepeda L.G. (2005). New *S,O*-acetals from (1*R*)-(−)-myrtenal as chiral auxiliaries in nucleophilic additions. Tetrahedron Lett..

[11] Verhe R., Courtheyn D., de Kimpe N., de Buyck L., Schamp N. (1982). Preparation of 1,1-Dialkoxy-2-alkanones. Synthesis.

[12] Tiecco M., Testaferri L., Tingoli M., Chianelli D., Bartoli D. (1991). Selenium-mediated conversion of alkynes into α-dicarbonyl compounds. J. Org. Chem..

[13] Tiecco M., Testaferri L., Tingoli M., Bartoli D. (1990). Selenium-catalyzed conversion of methyl ketones intoα-keto acetals. J. Org. Chem..

[14] Nair V., Nair L.G., Panicker S.B., Sheeba V., Augustine A. (2000). Novel Cerium(IV) Ammonium Nitrate Mediated Transformation of Styrenes to α-Methoxy Acetophenones. Chem. Lett..

[15] Wegner G., Karbach S., Smuda H., Hickmann E., Kober R., Seele R., Zierke T. (1992). Process for the preparation of a,a-dialkoxy ketones by treatment of aldehydes or ketones with alkyl nitrite. Eur. Pat. Appl. EP 472118 A1.

[16] Tang H., Chen S., Zhang P. (1985). (1.3)-Sigmatropic rearrangements of 1,3-dialkyloxy-acetylactones and-acetones. Huaxue Xuebao.

[17] Yu Y., Chen G., Zhu J., Zhang X., Chen S., Tang H., Zhang P. (1990). A study of rearrangement of some 1,3-dimethoxyalkan-2-ones. J. Chem. Soc. Perkin Trans. 1.

[18] Devos A., Remion J., Frisque-Hesbain A.-M., Colens A., Ghosez L. (1979). Synthesis of acyl halides under very mild conditions. J. Chem. Soc. Chem. Commun..

[19] Adamczyk M., Johnson D.D., Mattingly P.G., Pan Y., Reddy R.E. (2002). A convenient method for the preparation of α-ketoacetals. Synth. Comm..

[B20-molecules-17-13864] 20.These α-ketoacetals can be purchased from Sigma-Aldrich, Co., St. Louis, MO, USA.

[21] A very complete review concerning synthesis and use of Weinreb amides is highly recommendable: Balasubramaniam S., Aidhen I.S. (2008). The growing synthetic utility of the Weinreb amide. Synthesis.

[22] Nahm S., Weinreb S.M. (1981). *N*-methoxy-*N*-methylamides as effective acylating agents. Tetrahedron Lett..

[23] Williams J.M., Jobson R.B., Yasuda N., Marchesini G., Dolling U.H., Grabowski E.J.J. (1995). A new general method for preparation of *N*-Methoxy-*N*-methylamides. Application in direct conversion of an ester to a ketone. Tetrahedron Lett..

[24] Toda N., Ori M., Takami K., Tago K., Kogen H. (2003). Total Synthesis of (+)-Benzastatin E via Diastereoselective Grignard Addition to 2-Acylindoline. Org. Lett..

[25] Ki-Jong H., Misoo K. (2007). Direct Synthesis of Weinreb Amides from Carboxylic Acids Using Triphosgene. Lett. Org. Chem..

[B26-molecules-17-13864] 26.Rare Chemicals Catalogue 20461-86-3 and 3559-62. Supplier Name: Rare Chemicals GmbH, Catalog Publication Date: 6 June 2012.

[27] Graham S.L., Scholz T.H. (1990). A new mode of reactivity of *N*-methoxy-*N*-methylamides with strongly basic reagents. Tetrahedron Lett..

[28] Meester W.J.N., Van Dijk R., Van Maarseveen J.H., Rutjes F.P.J.T., Hermkens P.H.H., Hiemstra H. (2001). Highly diastereoselective synthesis of β-amino alcohols. J. Chem. Soc. Perkin Trans. 1.

[29] Azizi N., Saidi M.R. (2005). Highly Chemoselective Addition of Amines to Epoxides in Water. Org. Lett..

[30] Goswami S., Maity A.C., Fun H.-K., Chantrapromma S. (2009). The smallest vicinal tricarbonyl compound as a monohydrate and tetracarbonyl compound as a thiane derivative - first effective synthesis, characterization and chemistry. Eur. J. Org. Chem..

[B31-molecules-17-13864] 31.Groening, Carsten; Ebel, Klaus; Kaibel, Gerd; Therre, Joerg; Koopmann, Juergen; Menig, Helmuth; Fritz, Gerard; Dietz, Rainer. Preparation of methylglyoxal dimethyl acetal. Eur. Pat. Appl. EP 704422 A1, 3 April 1996.

[32] Guseinov F.I., Tagiev S.Sh., Moskva V.V. (1995). Reaction of α-Chloro- and α,α-Dichloro-β-oxo-substituted Aldehydes with Anionic Nucleophiles. Zh. Org. Khimii.

[33] Khamliche L., Bakkas S., Robert A. (1994). Selective protection of the functionalities of α-hydroxy unsaturated aldehydes. Synthesis.

[34] Devulapally R., Hon Y.-S. (2011). The first total synthesis of (±)-zenkequinone. Tetrahedron Lett..

[35] Wu C., Kawasaki K., Ohgiya S., Ohmiya Y. (2006). Syntheses and evaluation of the bioluminescent activity of (S)-Cypridina luciferin and its analogs. Tetrahedron Lett..

[36] Keiko N.A., Funtikova E.A., Stepanova L.G., Chuvashev Y.A., Larina L.I. (2002). Reactions of 2-Alkoxypropenals with Thiols in Neutral and Acid Media. Russ. J. Org. Chem..

[B37-molecules-17-13864] 37.Noack, Rainer; Palm, Clemens; Groening, Carsten; Lipowsky, Gunter. Preparation of hydroxymethyl-1,2-diphenyloxiranes. PCT. Int. Appl. WO 2010089353 A1 12 August 2010.

[38] Studer M. (2001). Production of optically active α-hydroxy acetals. PCT Int. Appl..

[39] Durham T.B., Hahn P.J., Kohn T.J., McCarthy J.R., Broughton H.B., Dally R.D., Gonzalez-Garcia M.R., Henry K.J., Shepherd T.A., Erickson J.A. (2006). Preparation of acylated 2-amino-1-(morpholin-3-yl)ethanols and derivatives as BACE inhibitors for treating Alzheimer’s. PCT Int. Appl..

[40] Bringmann G., Geisler J.P. (1989). A Simple, Chiral-Pool-Independent Synthesis of Enantiomerically Pure Alanine-Derived α-Amino Aldehyde Acetals. Synthesis.

[41] Panunzi B., Rotiroti L., Tingoli M. (2003). Solvent directed electrophilic iodination and phenylselenenylation of activated alkyl aryl ketones. Tetrahedron Lett..

[42] Nishio T., Omote Y. (1980). The substitution reaction of 2-aralkylthio-1-alkenyl and 2-alkylsulfinyl-1-alkenyl ketones with alkoxides: preparation of 2-alkoxy-1-alkenyl ketones. Synthesis.

[43] Bedore M.W., Zaborenko N., Jensen K.F., Jamison T.F. (2010). Aminolysis of Epoxides in a Microreactor System: A Continuous Flow Approach to β-Amino Alcohols. Org. Process Res. Dev..

[44] Chung J.Y. L., Cvetovich R., Amato J., McWilliams J., Reamer R., DiMichele L. (2005). Enantioselective Nitrile Anion Cyclization to Substituted Pyrrolidines. A Highly Efficient Synthesis of (3*S*,4*R*)-*N*-*tert*-Butyl-4-Arylpyrrolidine-3-Carboxylic Acid. J. Org. Chem..

[45] Alcaide B., Escobar G., Perez-Ossorio R., Plumet J., Sanz D. (1984). The reaction of phenylglyoxal with primary aliphatic and aromatic amines. Synthesis of phenylglyoxal monoimines and some derivatives. J. Chem. Res. Synop..

[46] Huerta G., Contreras‐Ordoñez G., Alvarez‐Toledano C., Santes V., Gómez E., Toscano R.A. (2004). Facile Synthesis of Aminoalcohols by Ring Opening of Epoxides Under Solvent Free Conditions. Synth. Commun..

[47] Maeda H., Furuyoshi S., Nakatsuji Y., Okahara M. (1983). Synthesis of monoaza crown ethers from *N,N*-bis[oligo(oxyalkylene)]amines and oligoethylene glycol bis(p-toluenesulfonates) or corresponding dichlorides. Bull. Chem. Soc. Jap..

[48] Negrón-Silva G., Hernández-Reyes C.X., Ángeles-Beltrán D., Lomas-Romero L., González-Zamora E., Méndez-Vivar J. (2007). Comparative Study of Regioselective Synthesis of β-Aminoalcohols under Solventless Conditions Catalyzed by Sulfated Zirconia and SZ/MCM-41. Molecules.

[49] Desai H., D’Souza B.R., Foether D., Johnson B.F., Lindsay H.A. (2007). Regioselectivity in a highly efficient, microwave-assisted epoxide aminolysis. Synthesis.

[50] Bonollo S., Fringuelli F., Pizzo F., Vaccaro L. (2006). A green route to β-amino alcohols via the uncatalyzed aminolysis of 1,2-epoxides by alkyl- and arylamines. Green Chem..

[51] Placzek A.T., Donelson J.L., Trivedi R., Gibbs R.A., De S.K. (2005). Scandium triflate as an efficient and useful catalyst for the synthesis of β-amino alcohols by regioselective ring opening of epoxides with amines under solvent-free conditions. Tetrahedron Lett..

[52] Munson H.R., Tankersley R.W. (1989). Substituted dialkanolamines,sulfur analogs and condensed 1,4-oxazine derivatives thereof in viral disease treatment. U.S. Patent.

[53] Cox P.J. (2003). 4-Bromo-2-(hydroxymethyl) phenol: helical hydrogen bonding, R22(12) rings and C-H···π interactions. Acta Cryst. Sect. C: Cryst. Struct. Commun..

[54] Bajwa N., Jennings M. (2006). Efficient and Selective Reduction Protocols of the 2,2-Dimethyl-1,3-benzodioxan-4-one Functional Group to Readily Provide Both Substituted Salicylaldehydes and 2-Hydroxybenzyl Alcohols. J. Org. Chem..

[55] Gisch N., Balzarini J., Meier C. (2007). 5-Diacetoxymethyl-cycloSal-d4TMP-A prototype of enzymatically activated cycloSal-pronucleotides. Nucleos. Nucleot. Nucl. Acids.

[56] Ding Y.S., Shiue C.Y., Fowler J.S., Wolf A.P., Plenevaux A. (1990). No-carrier-added (NCA) aryl [18F]fluorides via the nucleophilic aromatic substitution of electron-rich aromatic rings. J. Fluorine Chem..

[57] Aggarwal K., Esquivel-Zamora B. (2002). Application of the Chiral Acyl Anion Equivalent, trans-1,3-Dithiane 1,3-Dioxide to an Asymmetric Synthesis of (R)-Salbutamol. J. Org. Chem..

