# Supplementary material for: Efficient Preparation of α-Ketoacetals
Source: Molecules. 2012 Nov 22;17(12):13864–78. doi: 10.3390/molecules171213864 (PMC6268805; doi:10.3390/molecules171213864)

Supplementary Information

Figure S1. ^{1}H-NMR spectrum of Weinreb amide **2a**.

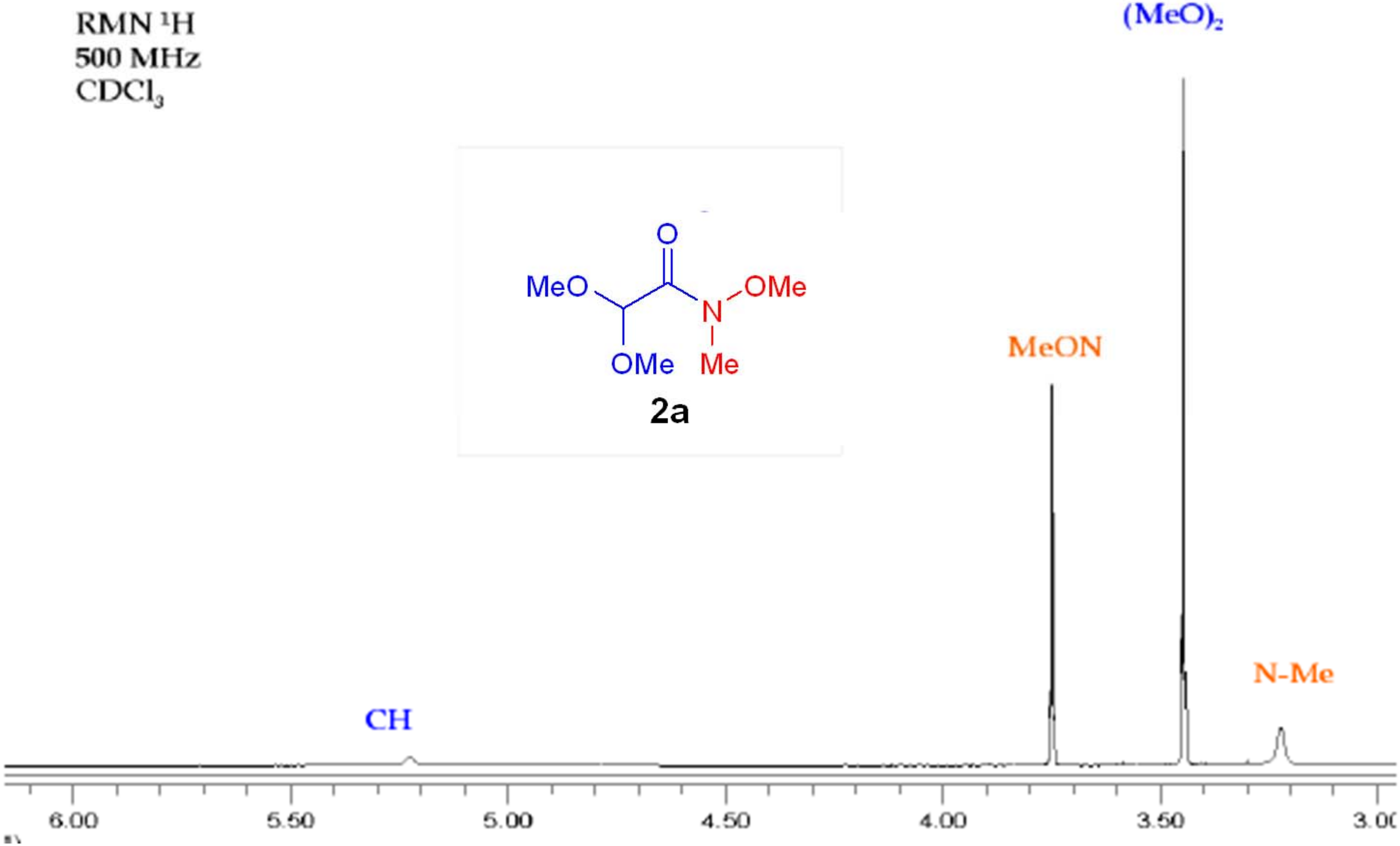

Figure S2. ^{13}C-NMR spectrum of Weinreb amide **2a**.

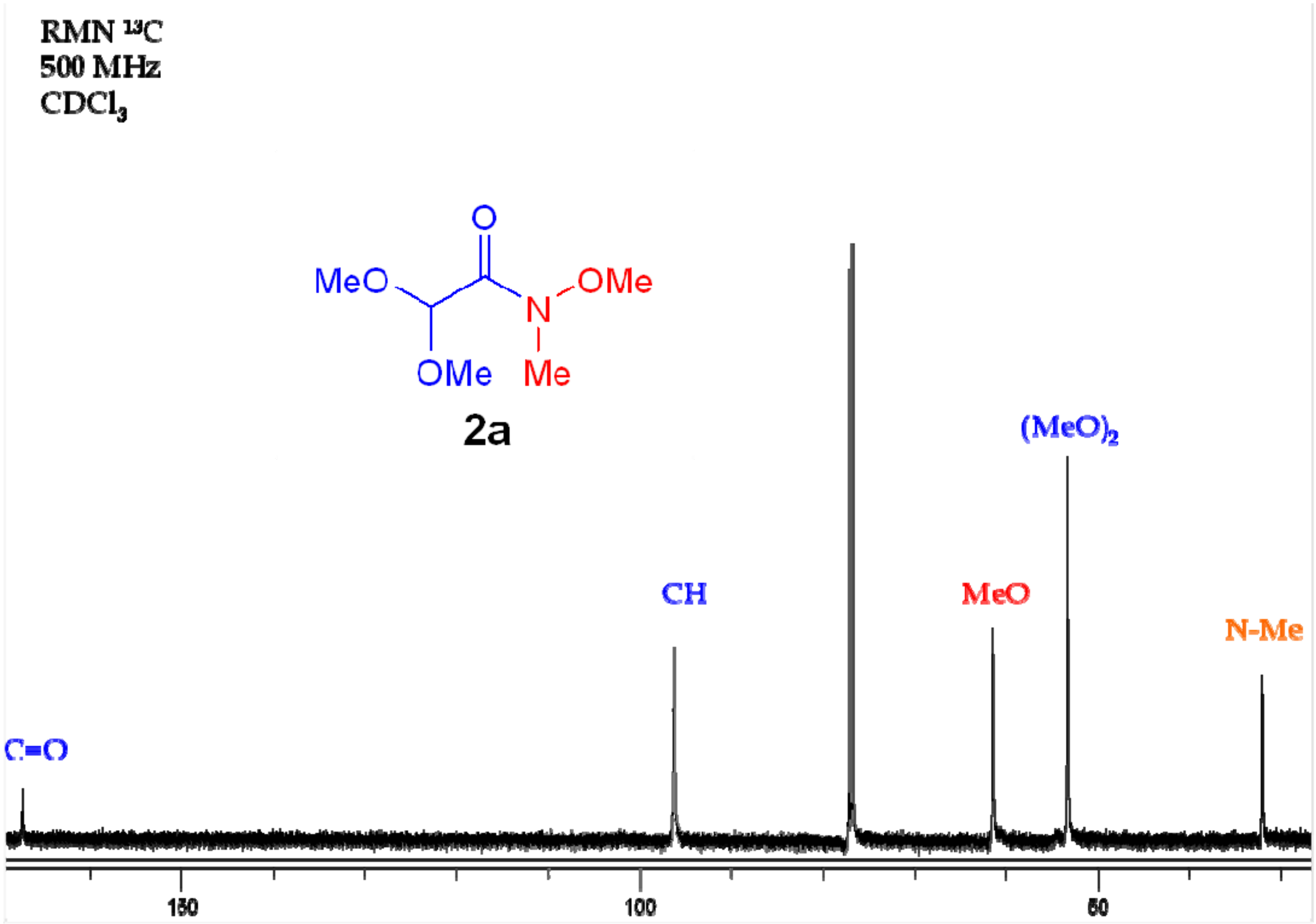

Figure S3. IR spectrum of Weinreb amide **2a**.

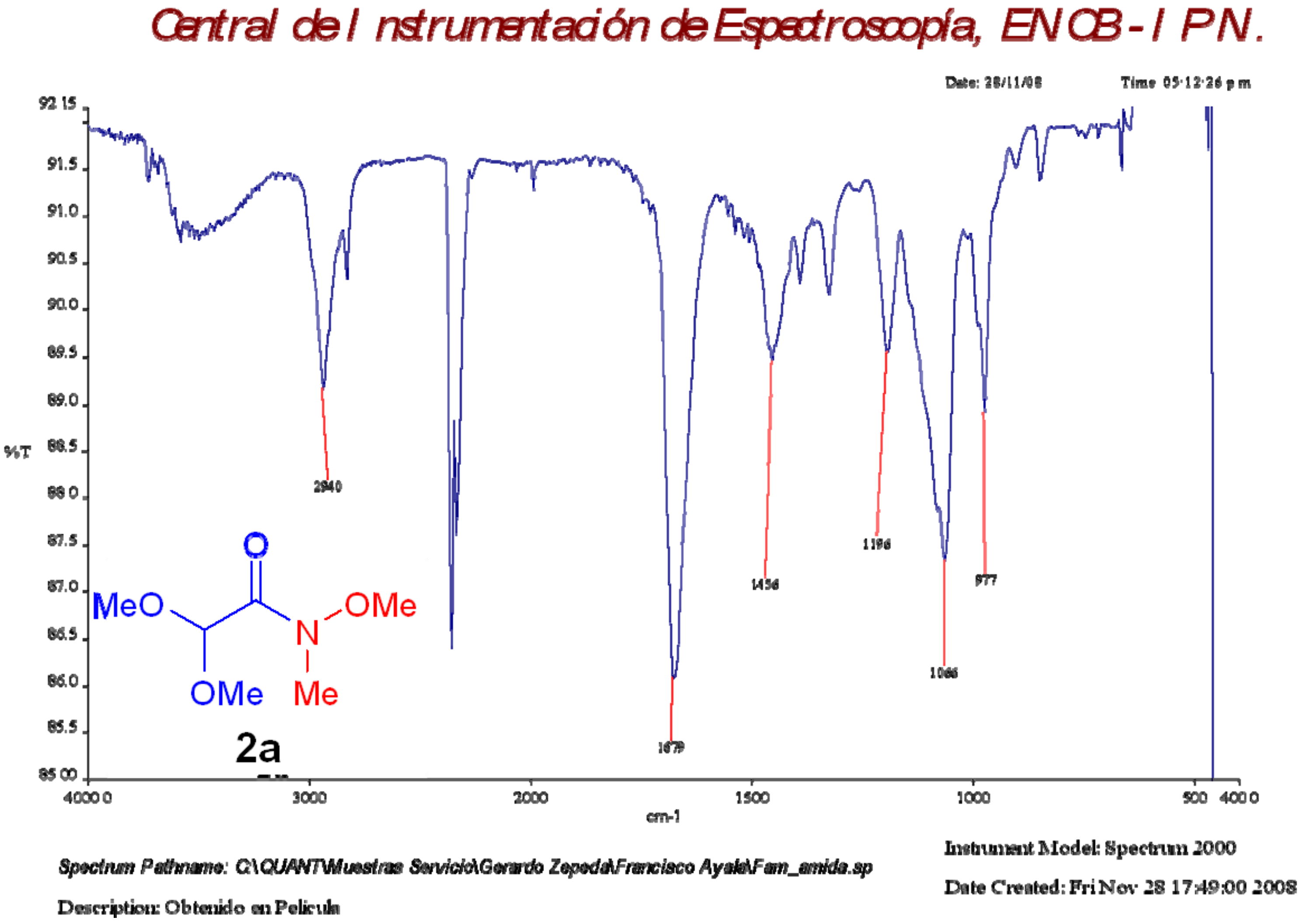

Figure S4. HR-EIMS spectrum of Weinreb amide **2a**.

IPN
11/10/2010 **Page 1**

File: GZ-FAM-Amida Date Run: 09-22-2009 (Time Run: 13:18:51)
Sample: Description
Instrument: JEOL GCmate
Inlet: Direct Probe **Ionization mode: EI+**

Scan: 5-12 **R.T.: .1**
Base: m/z 169; 3%FS TIC: 715856 **#Ions: 87**

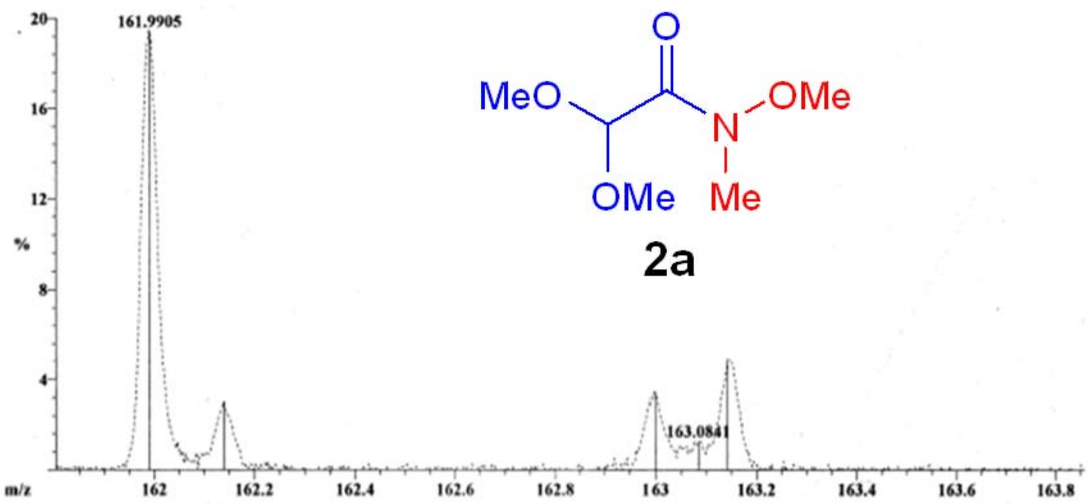

Selected Isotopes : C H N_1O_4 Error Limit : 50 ppm

Measured Mass	% Base	Formula	Calculated Mass	Error
163.0841	1.3%	$C_6H_{13}NO_4$	163.0845	-2.2

Figure S5. ^{1}H-NMR spectrum of Weinreb amide **2b**.

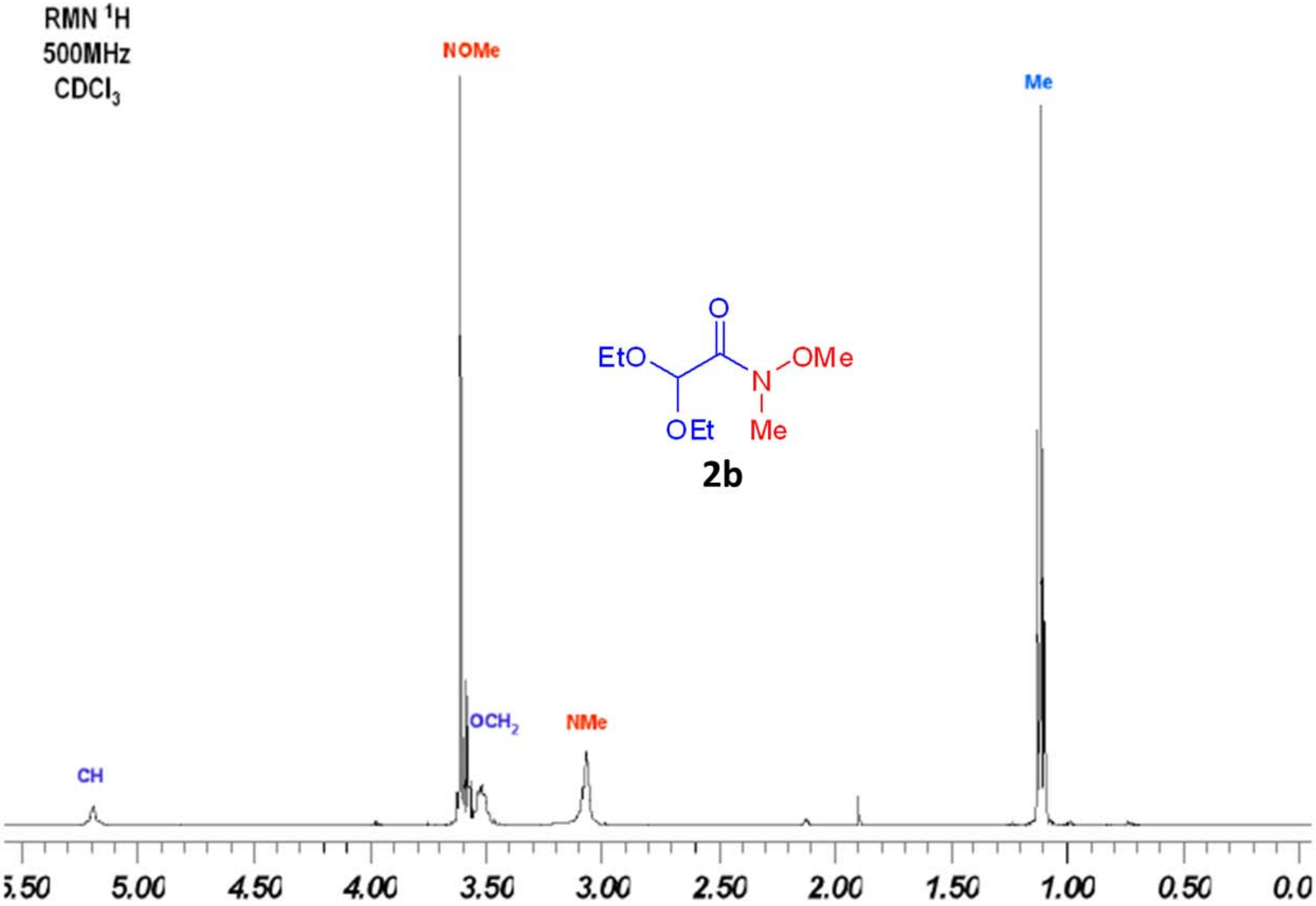

Figure S6. ^{13}C-NMR spectrum of Weinreb amide **2b**.

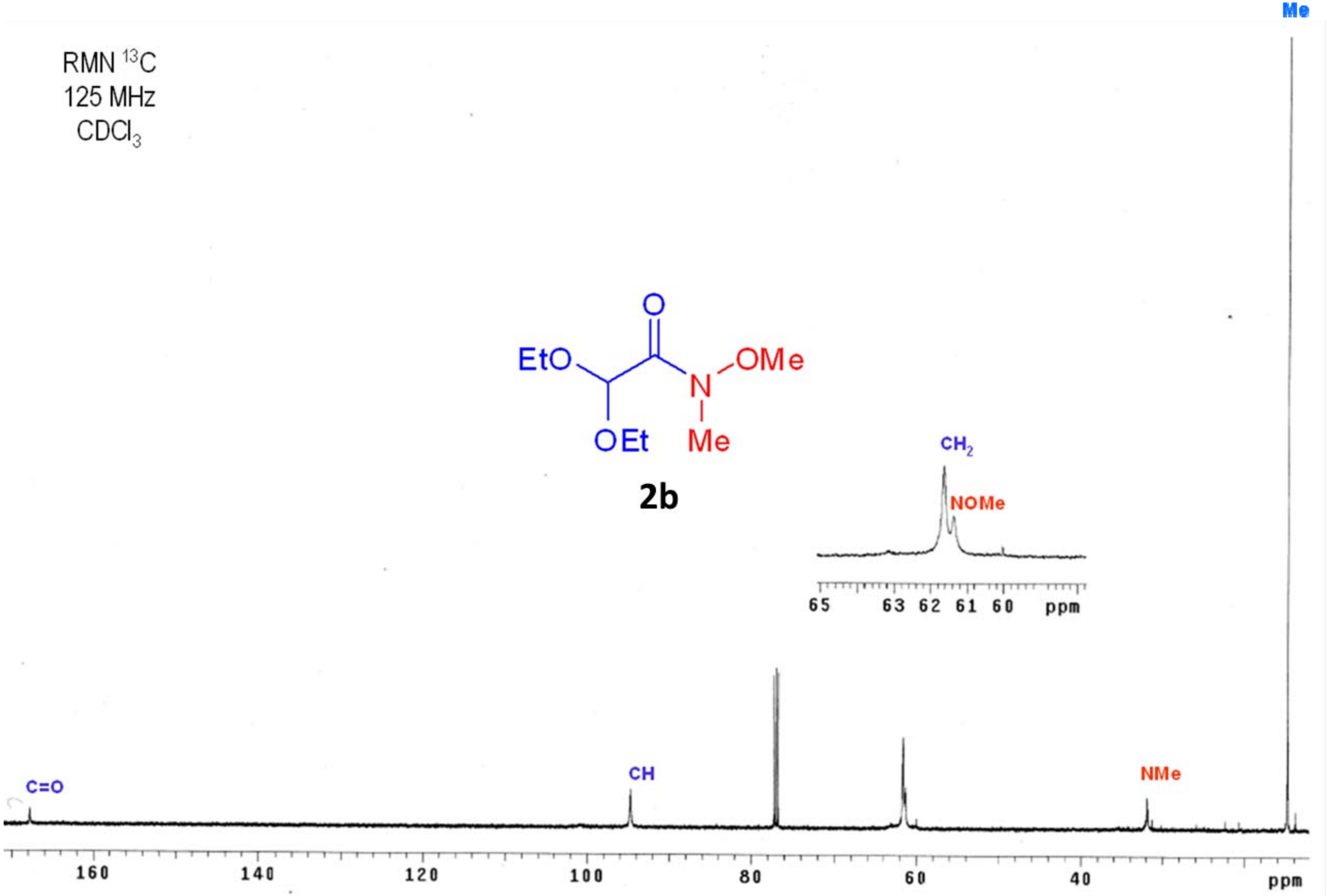

Figure S7. IR spectrum of Weinreb amide **2b**.

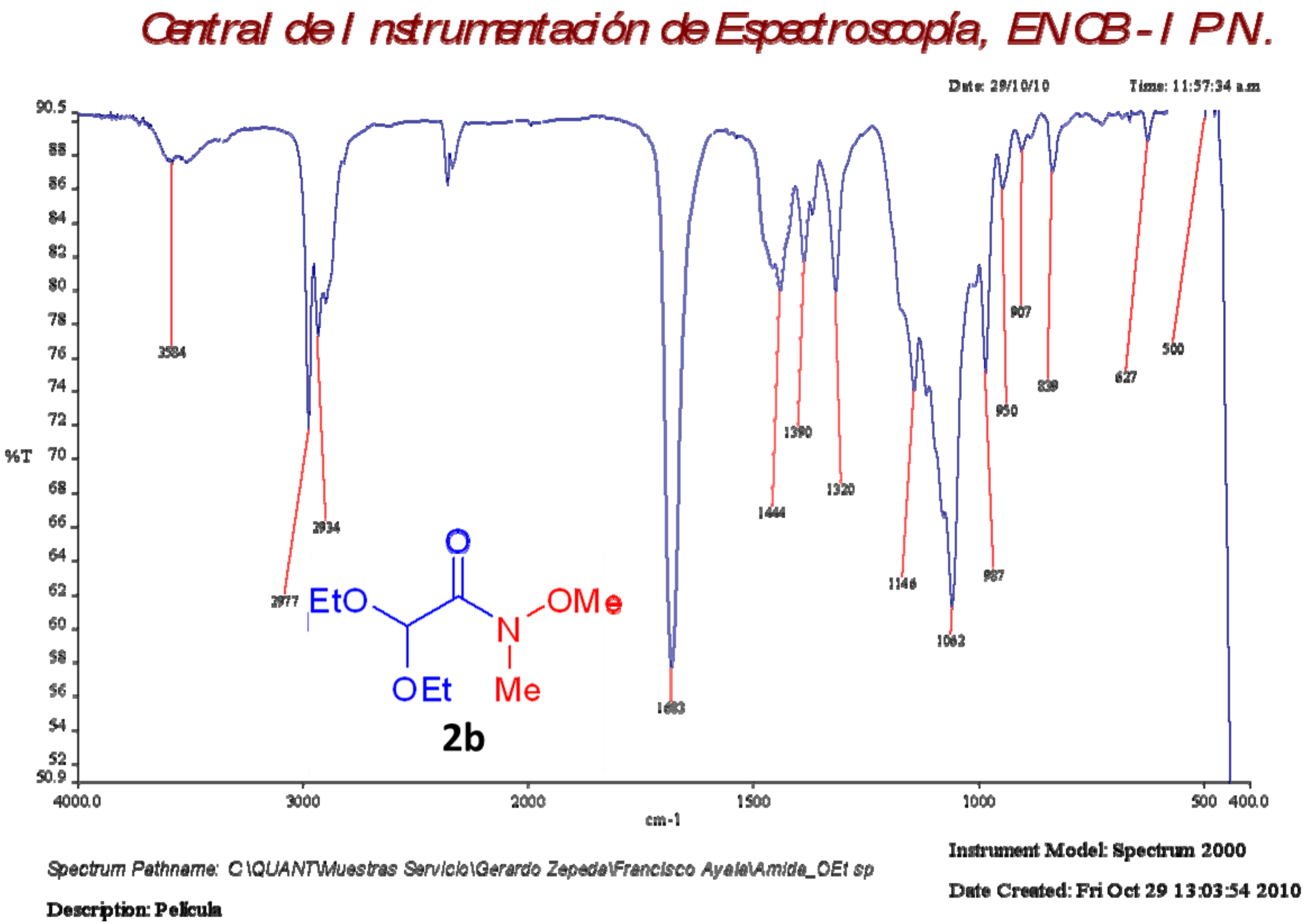

Figure S8. HR-EIMS spectrum of Weinreb amide **2b**.

IPN
11/10/2010 Page 1

File: GZ_FAM_AMIDA_ETODate Run: 10-06-2010 (Time Run: 11:25:14)
Sample: Amida weinreb etoxilo
Instrument: JEOL GCmate
Inlet: Direct Probe **Ionization mode: EI+**

Scan: 20 **R.T.: .23**
Base: m/z 167; .7%FS TIC: 431408 **#Ions: 827**

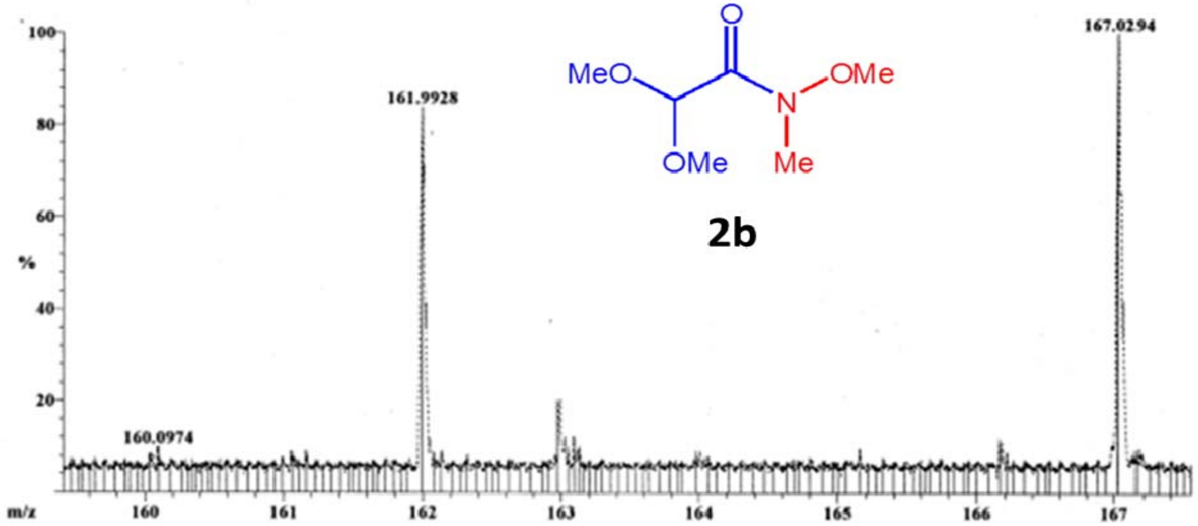

Selected Isotopes : C H N_1O_3 Error Limit : 50 ppm

Measured Mass	% Base	Formula	Calculated Mass	Error
160.0974	9.8%	$C_7H_{14}NO_3$	160.0974	0.2

Figure S9. ^{1}H-NMR spectrum of α-ketoacetal **3e**.

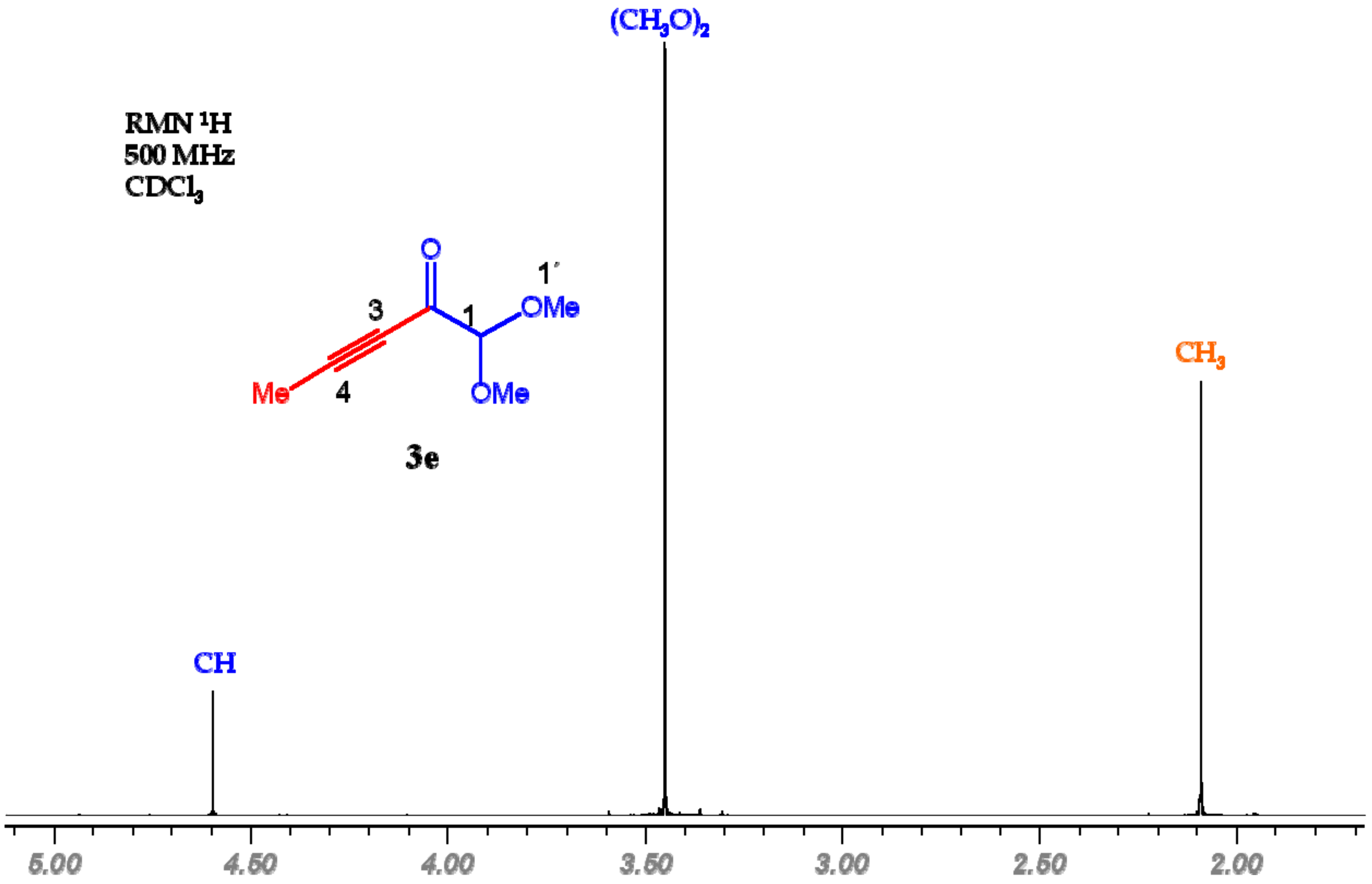

Figure S10. ^{13}C-NMR spectrum of *α*-ketoacetal **3e.**

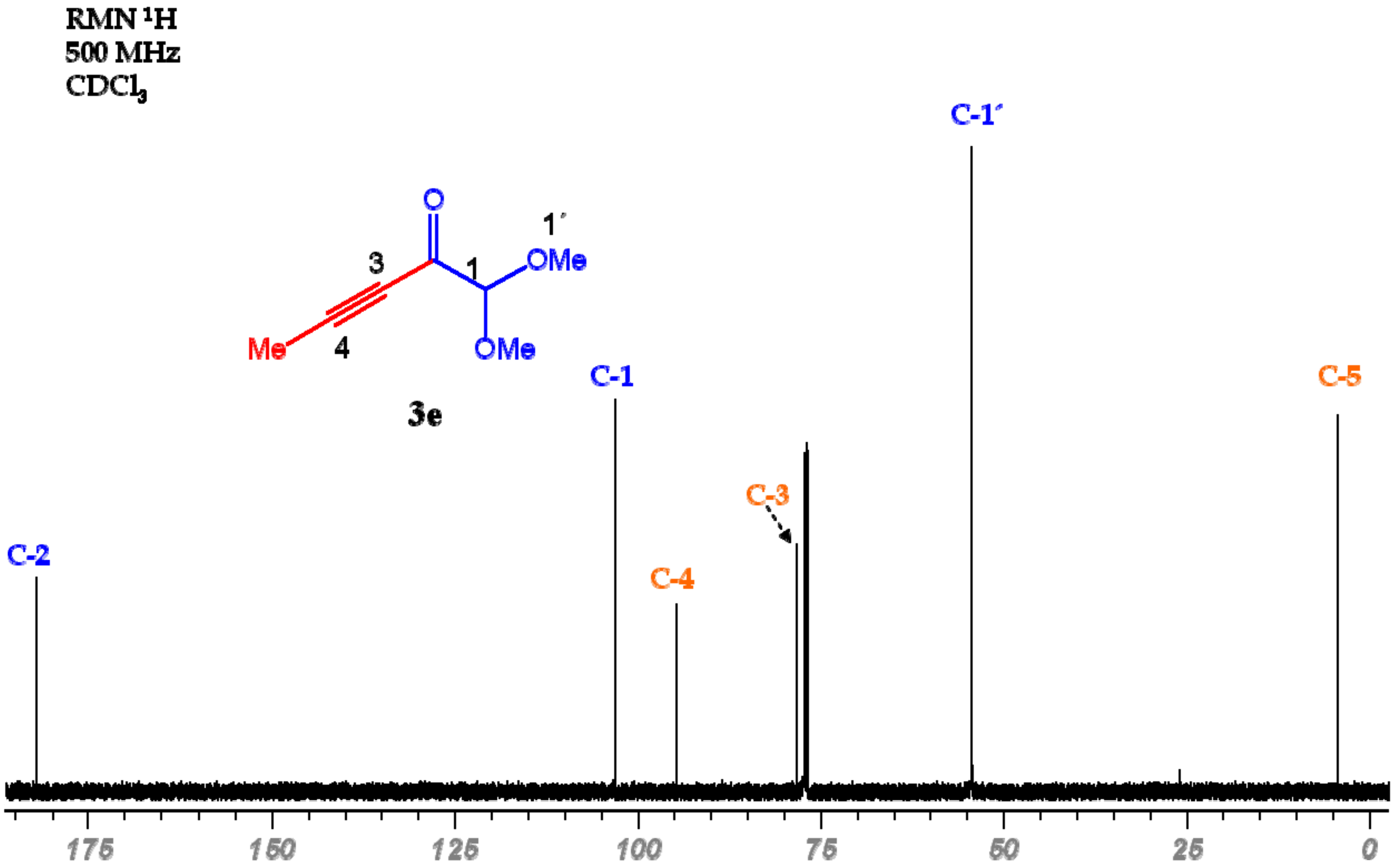

Figure S11. IR spectrum of α-ketoacetal **3e.**

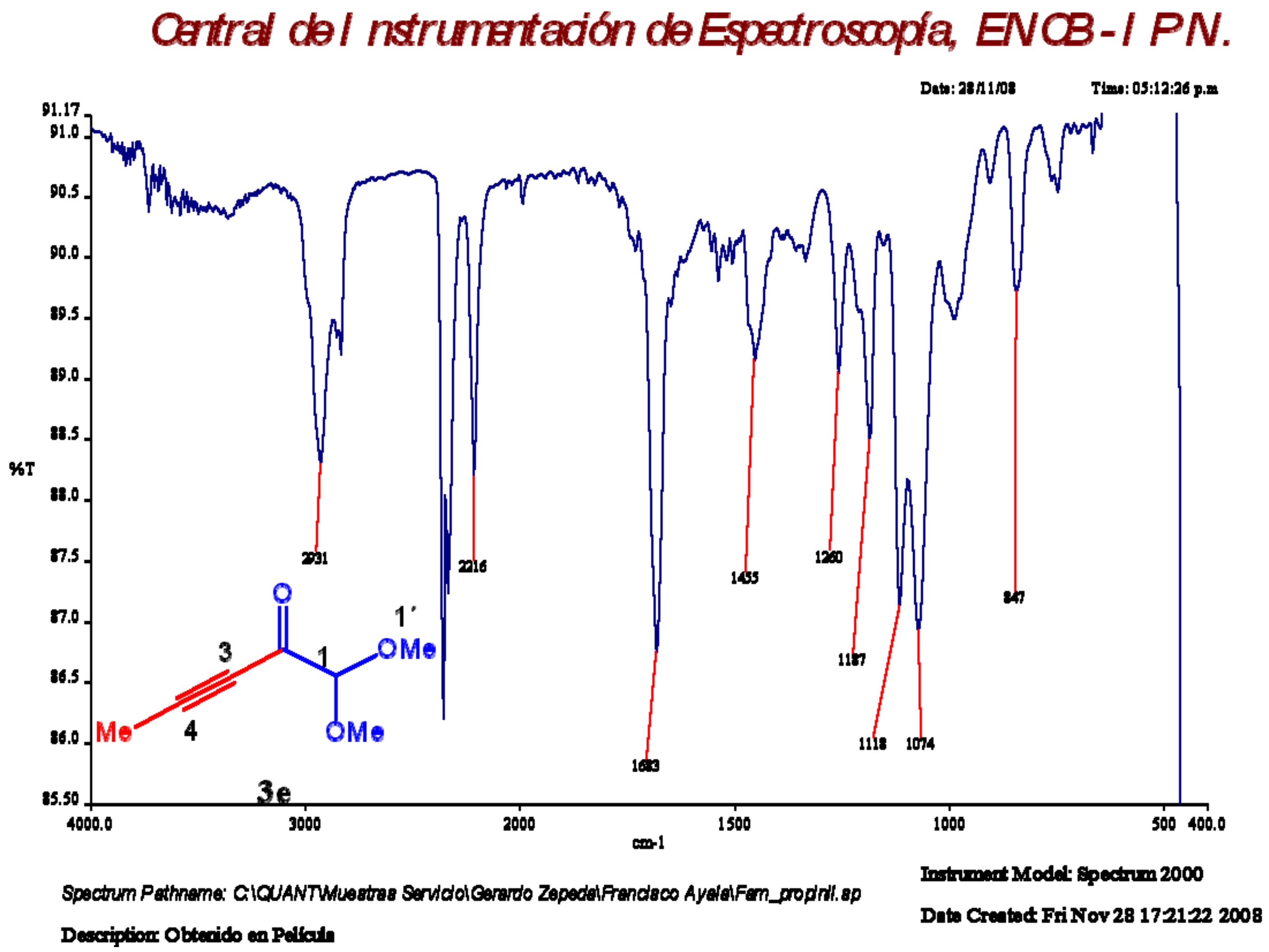

Figure S12. HR-EIMS spectrum of α-ketoacetal **3e.**

IPN
1/12/2009 **Page 1**

File: Coco_propinil_142Date Run: 01-12-2009 (Time Run: 18:53:23)
Sample: pm 142, propinil
Instrument: JEOL GCmate
Inlet: Direct Probe **Ionization mode: EI+**

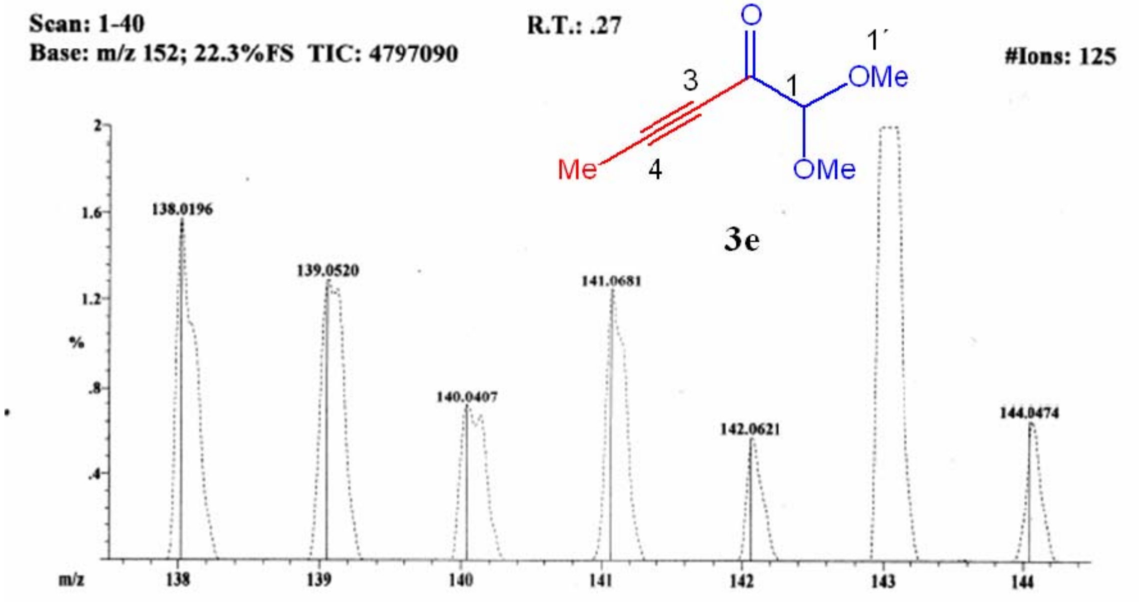

ı : 5-38 TIC : 5555787 Base : 26.2%FS #ions : 128

Selected Isotopes : $H_{0-12}C_{0-9}O_{0-4}$ Error Limit : 1 mmu

Measured Mass	*% Base*	*Formula*	*Calculated Mass*	*Error*
142.0621	0.6%	$C_7H_{10}O_3$	142.0630	-0.9

Figure S13. ^{1}H NMR spectrum of α-ketoacetal **3f.**

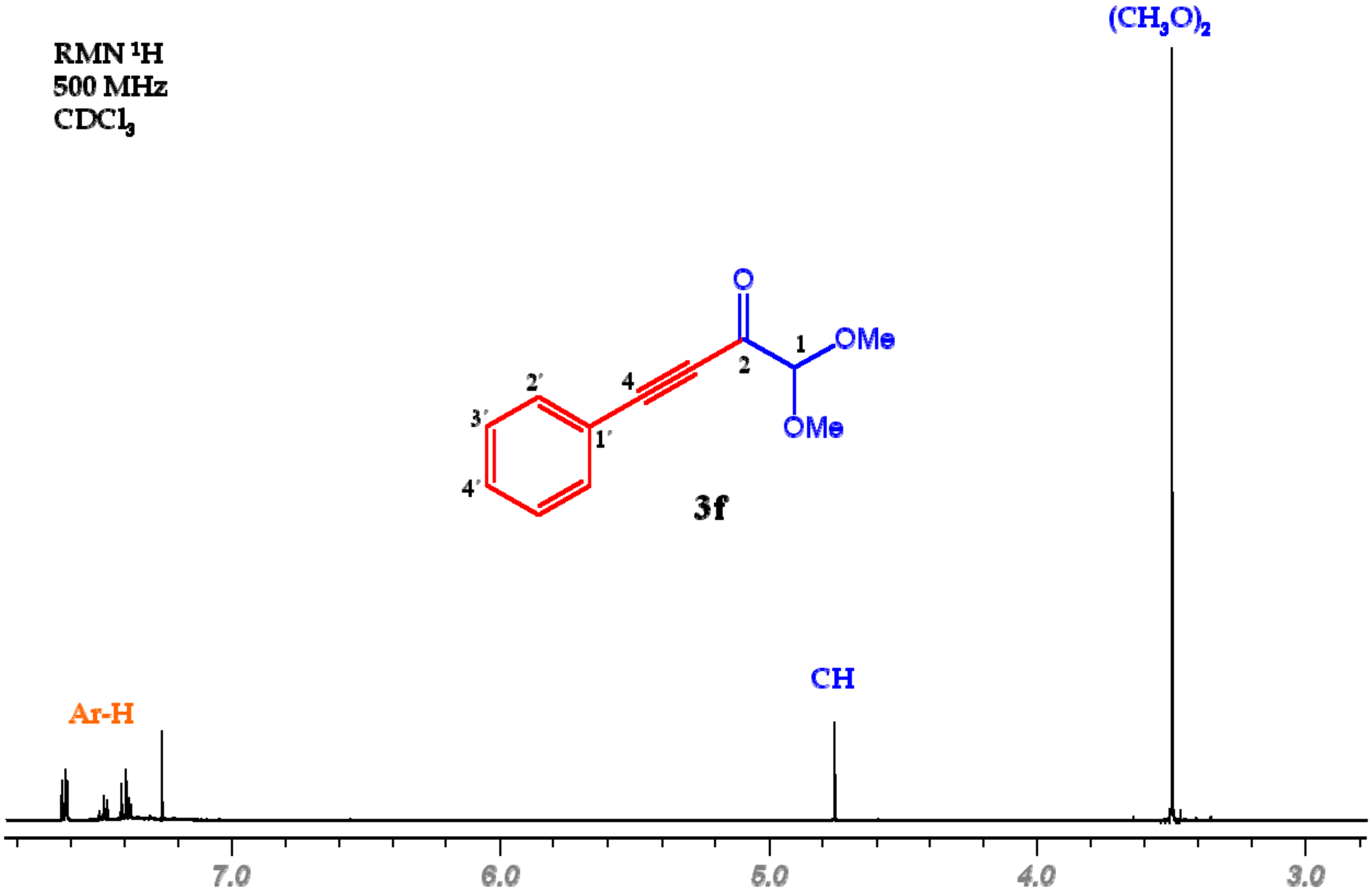

Figure S14. ^{13}C-NMR spectrum of α-ketoacetal **3f.**

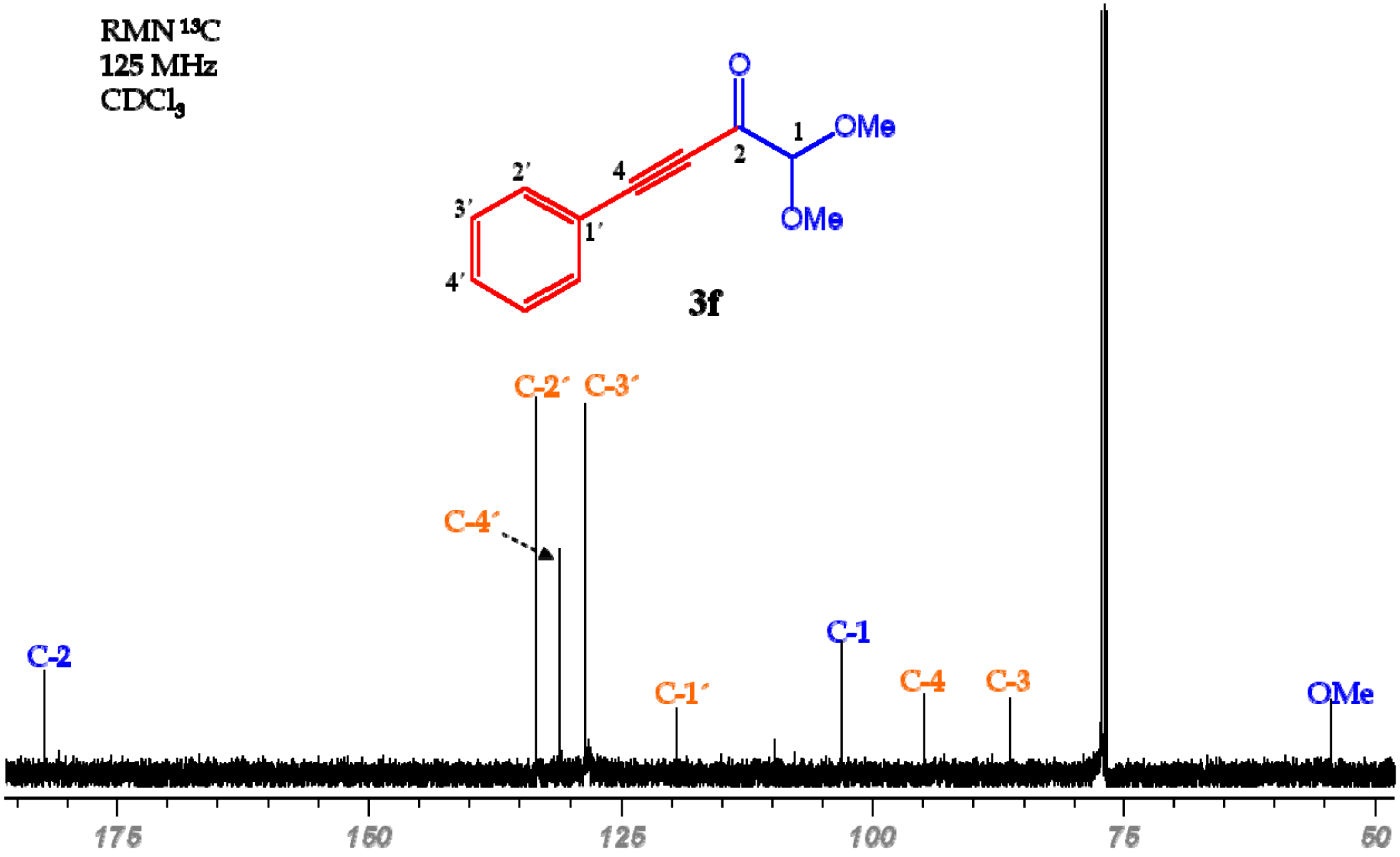

Figure S15. IR spectrum of α-ketoacetal **3f**.

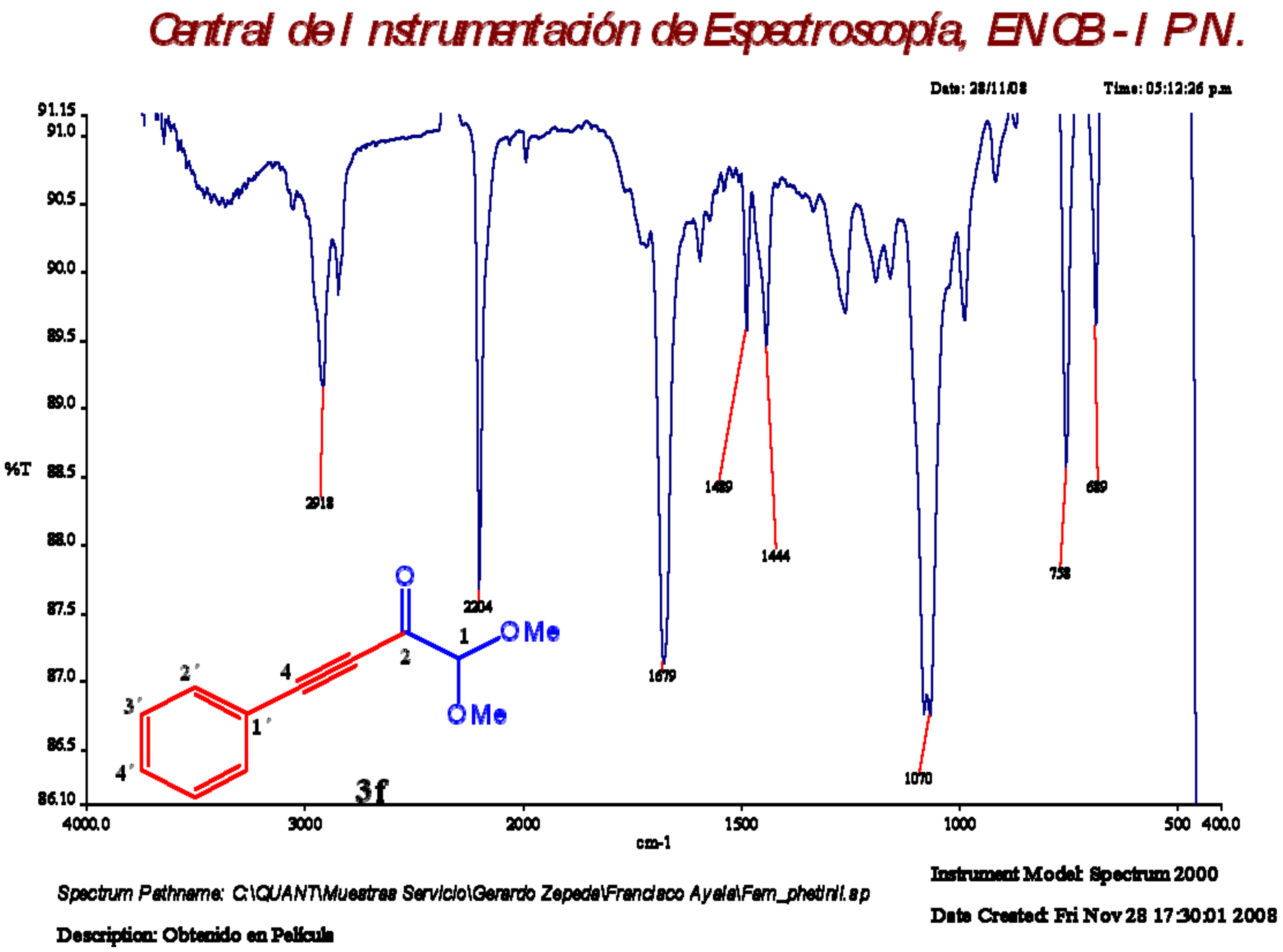

Figure S16. HR-EIMS spectrum of α-ketoacetal **3f**.

IPN
1/12/2009 Page 1

File: Coco_feniletinil Date Run: 01-12-2009 (Time Run: 20:08:22)
Sample: pm=204.222
Instrument: JEOL GCmate
Inlet: Direct Probe Ionization mode: EI+

Scan: 5-29 R.T.: .33
Base: m/z 181; .6%FS TIC: 237007 #Ions: 106

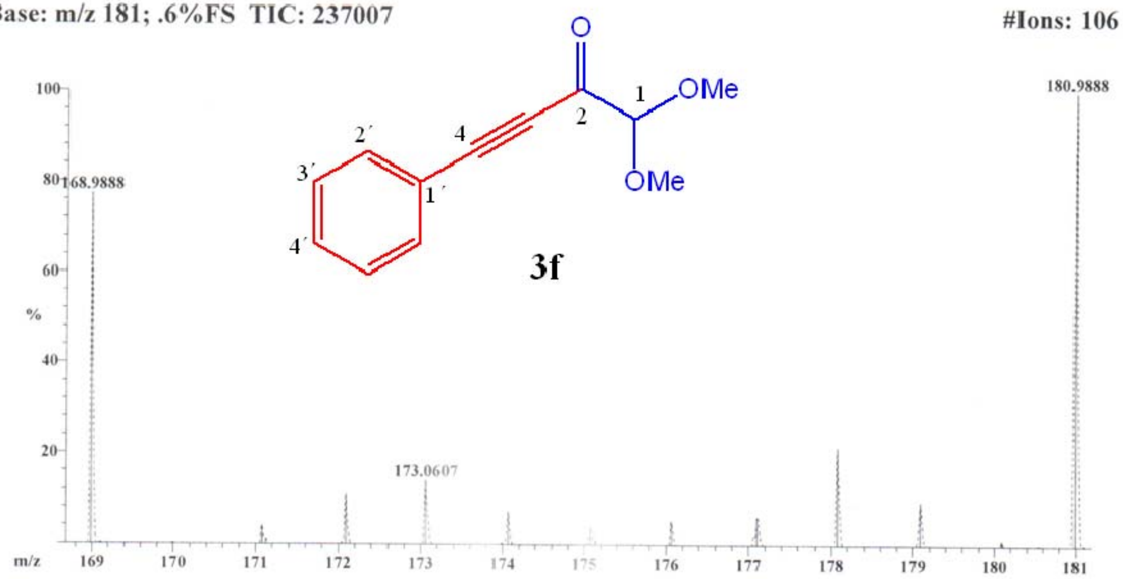

Selected Isotopes : $H_{0-15}C_{0-15}O_{0-4}$ Error Limit : 1 mmu

Measured Mass	% Base	Formula	Calculated Mass	Error
173.0607	13.8%	$C_{11}H_9O_2$	173.0603	0.4

Figure S17. ^{1}H-NMR spectrum of α-aminoalcohol **7a**.

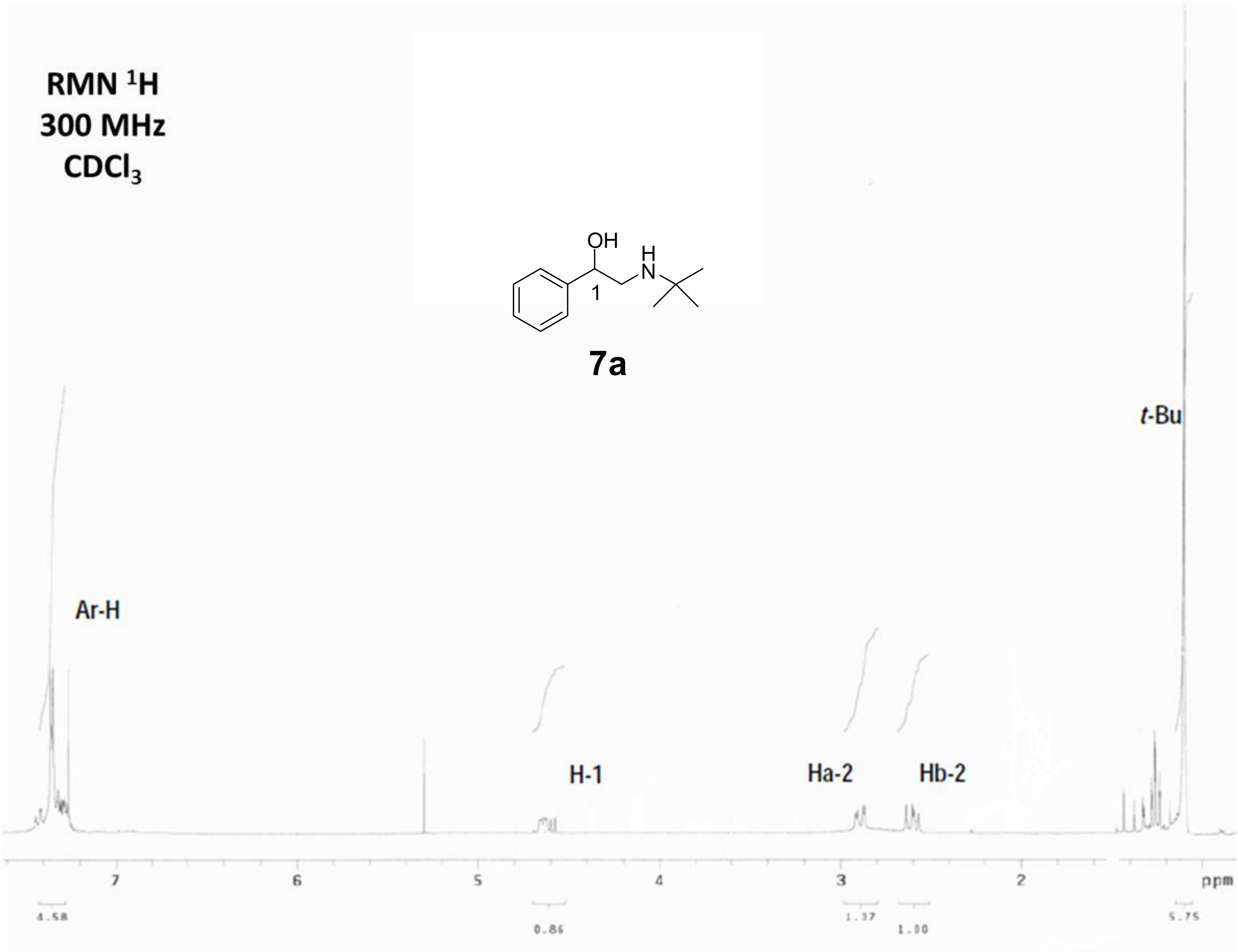

Figure S18. ¹H-NMR spectrum of α-aminoalcohol **7b**.

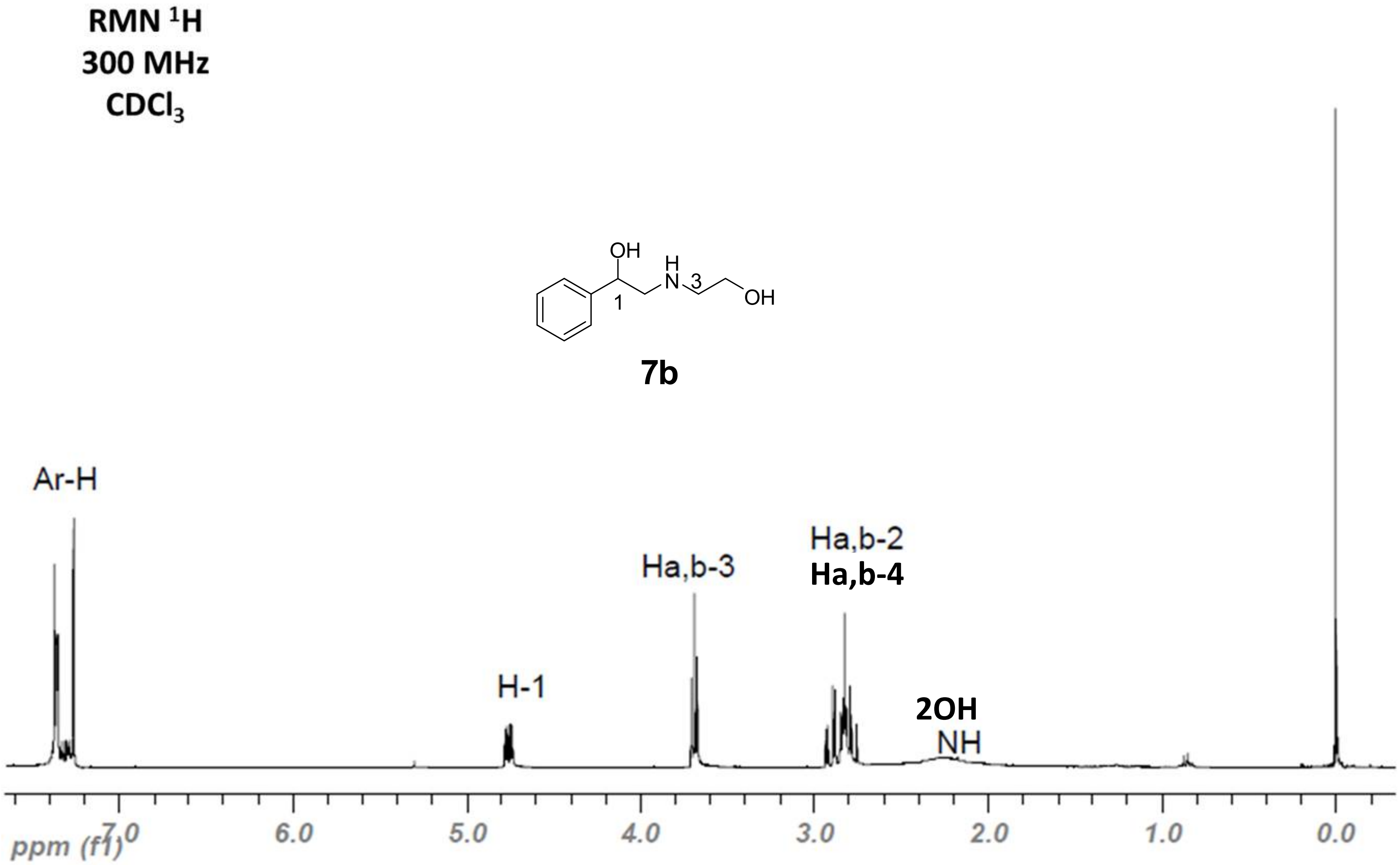

Figure S19. ^{1}H NMR spectrum of α-aminoalcohol **7c**.

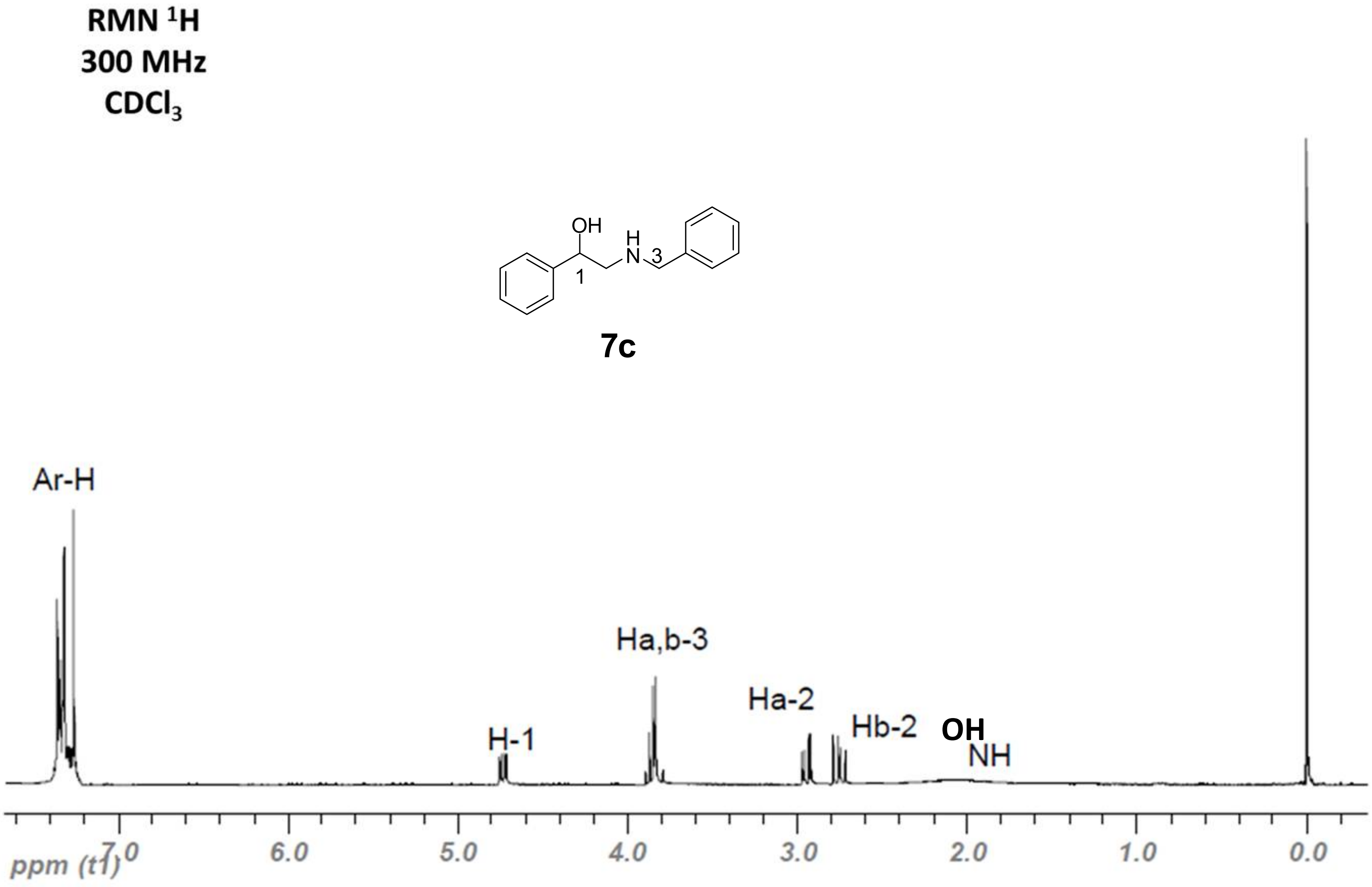

Figure S20. ^{1}H-NMR spectrum of α-aminoalcohol **7d**.

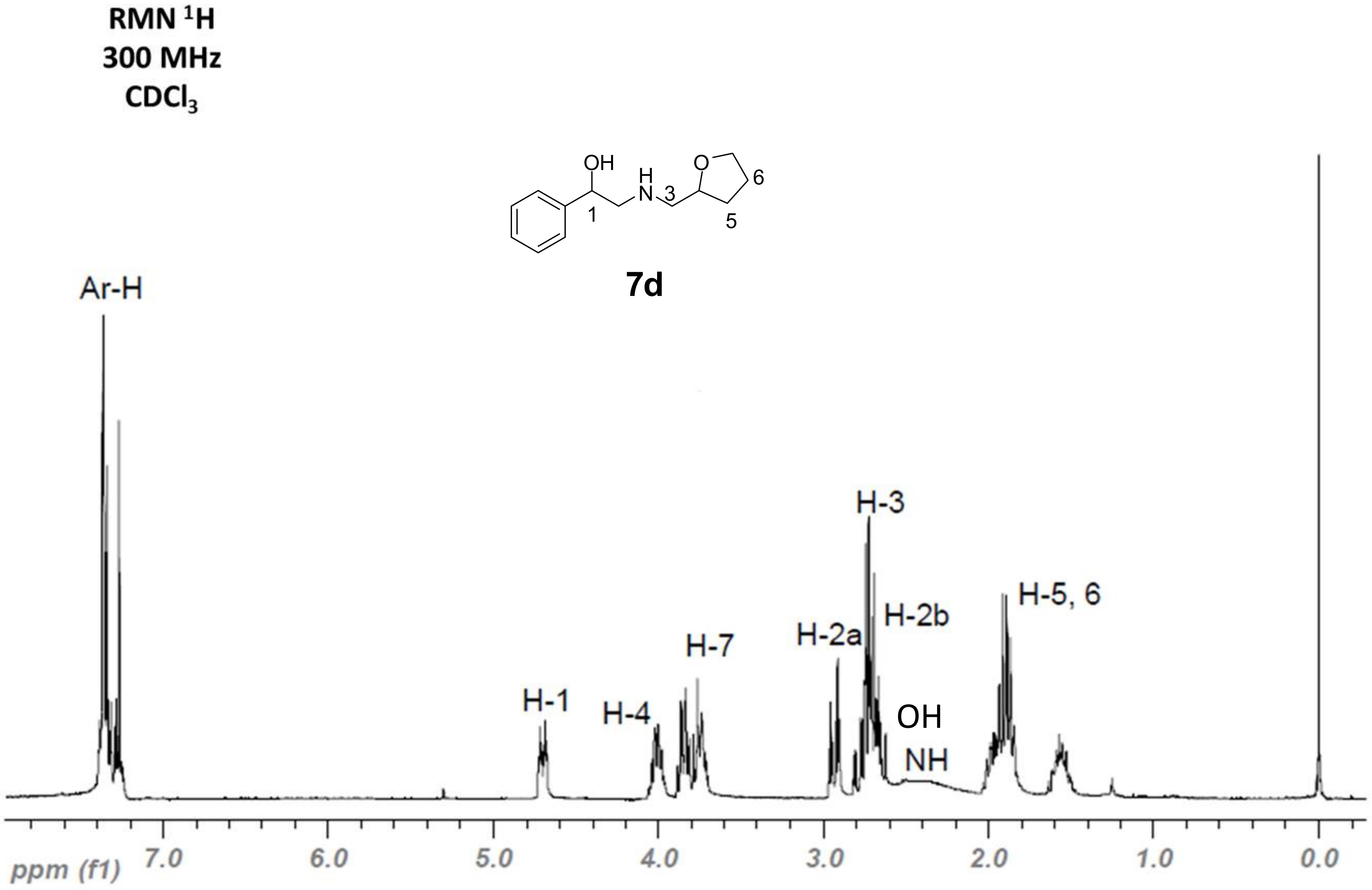

Figure S21. ^{1}H-NMR spectrum of bromo-2-(hydroxymethyl)phenol **8a**.

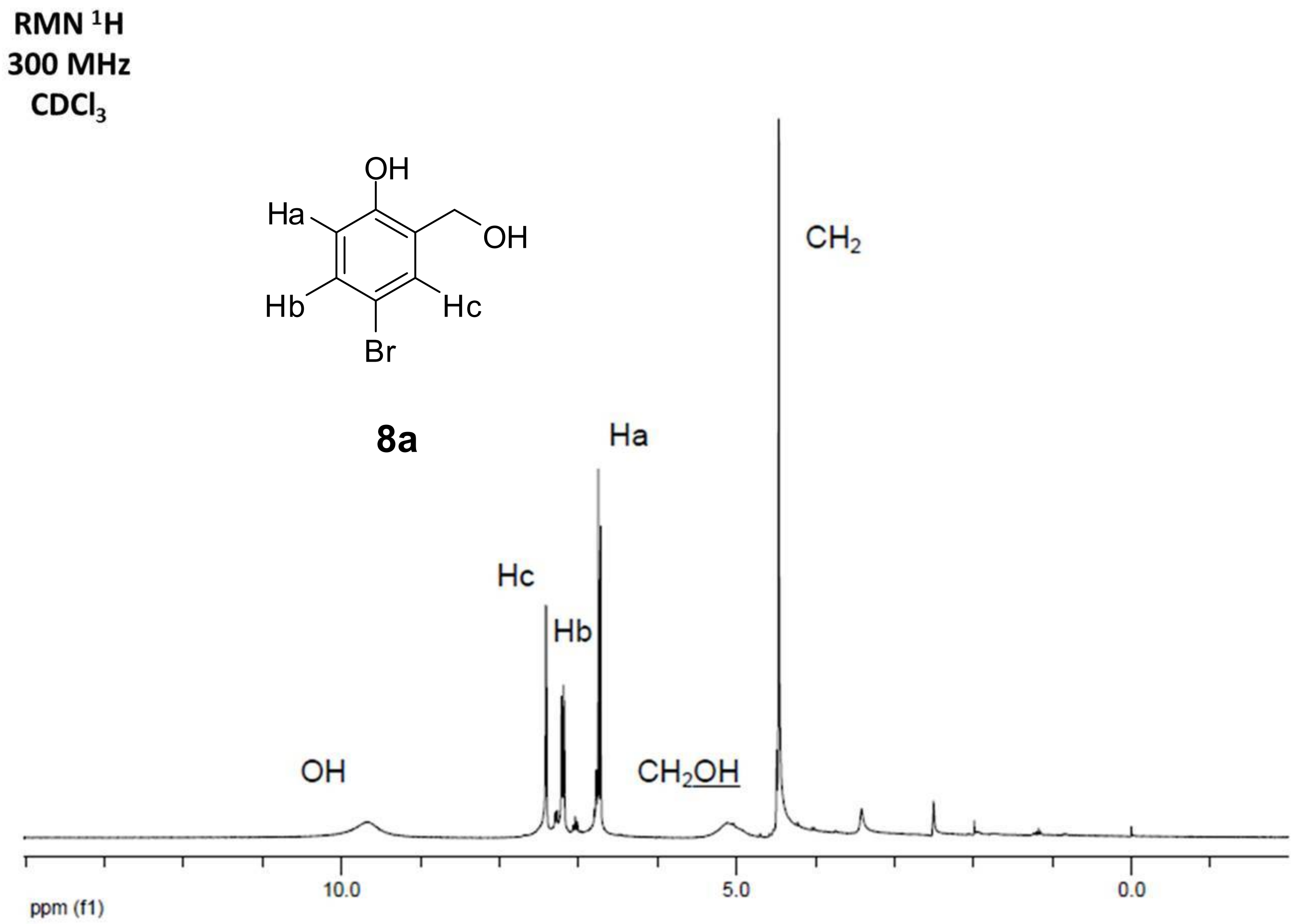

Figure S22. ^{1}H-NMR spectrum of benzodioxane **9**.

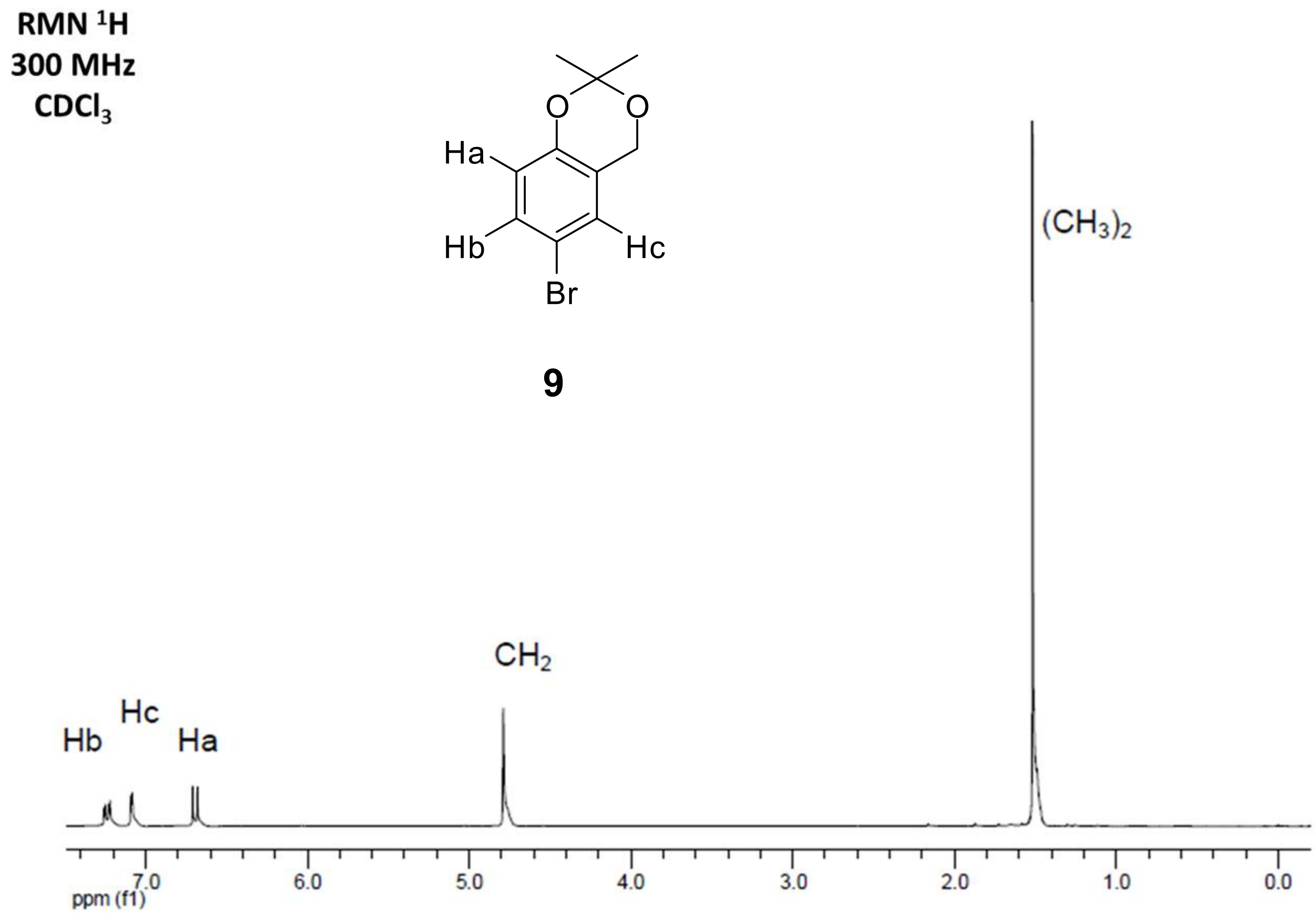

Figure S23. ^{1}H-NMR spectrum of α-ketoacetal **10**.

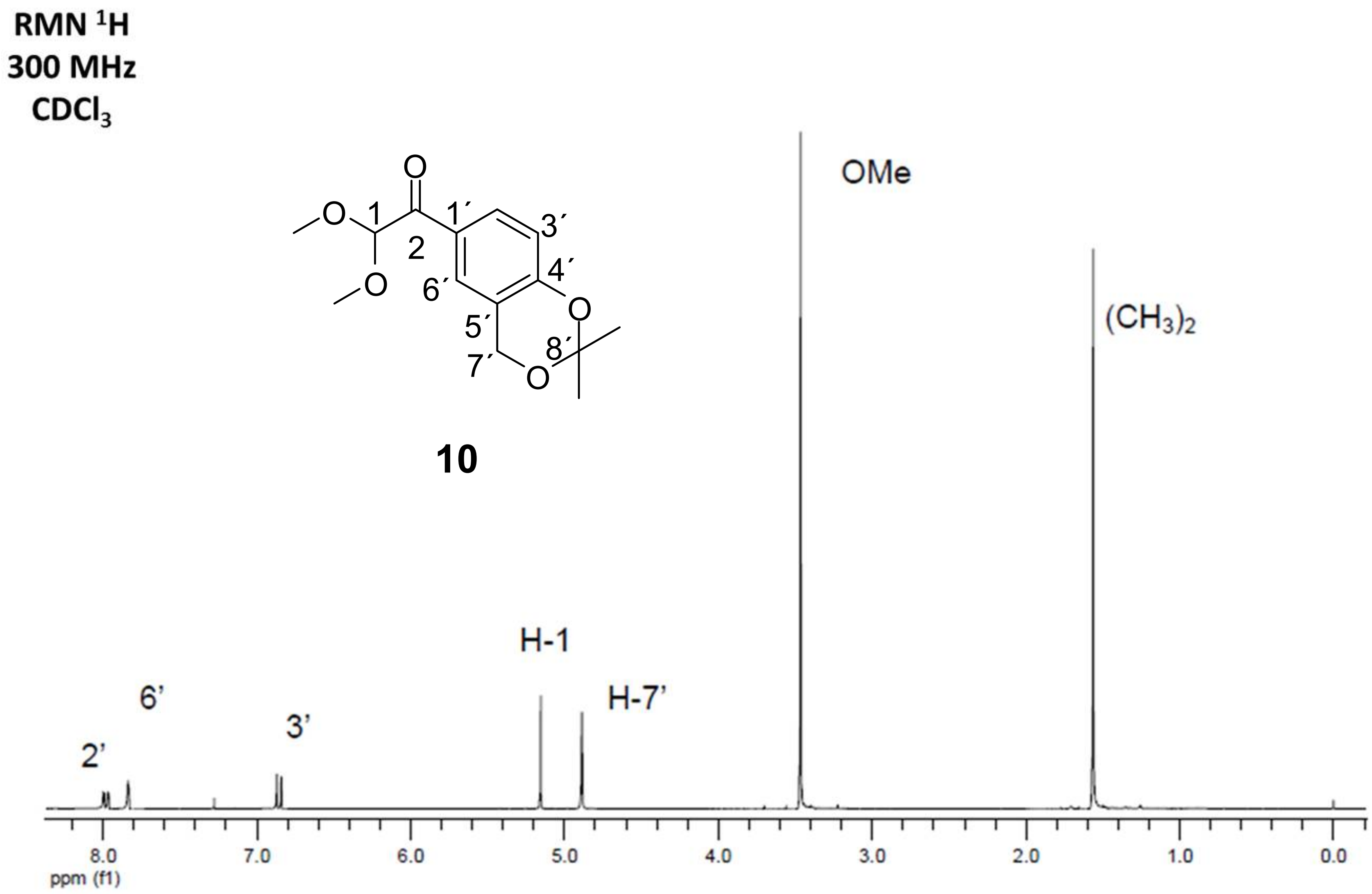

Figure S24. ^{13}C-NMR spectrum of α-ketoacetal **10**.

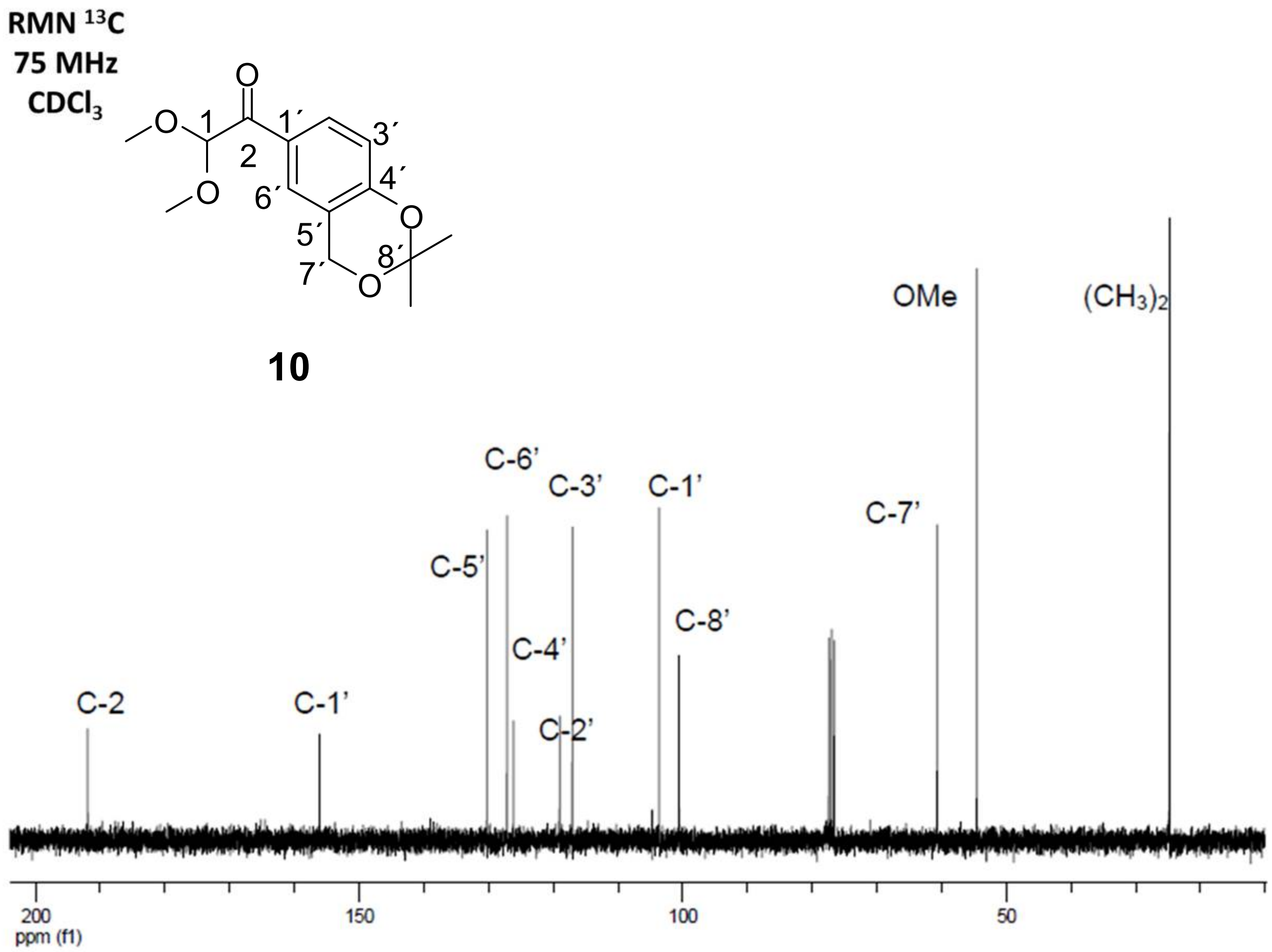

Figure S25. ^{13}H-NMR spectrum of α-hydroxyacetal **11**.

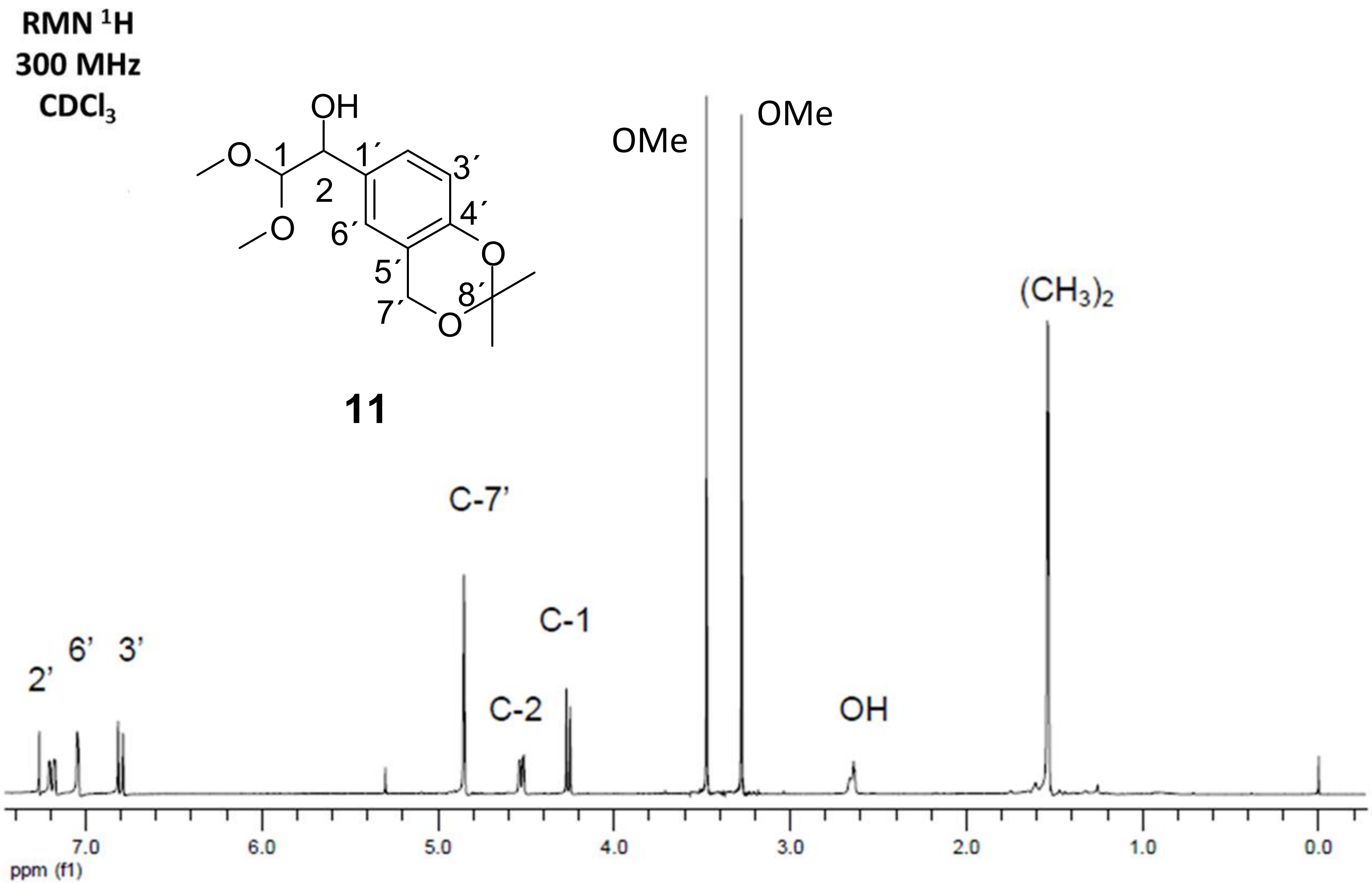

Figure S26. ^{1}H-NMR spectrum of *rac*-salbutamol.

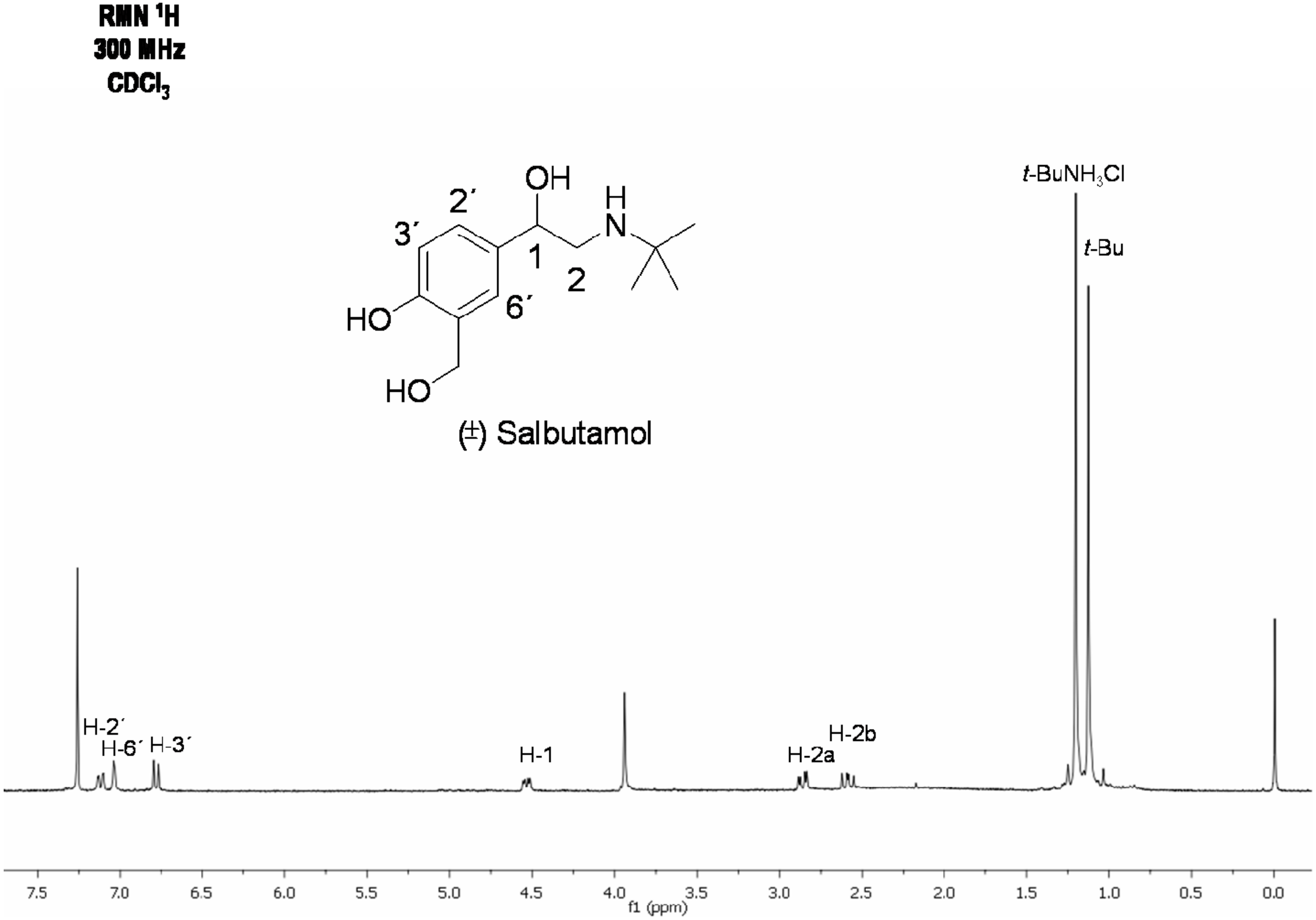

Figure S27. ^{13}C-NMR spectrum of *rac*-salbutamol.

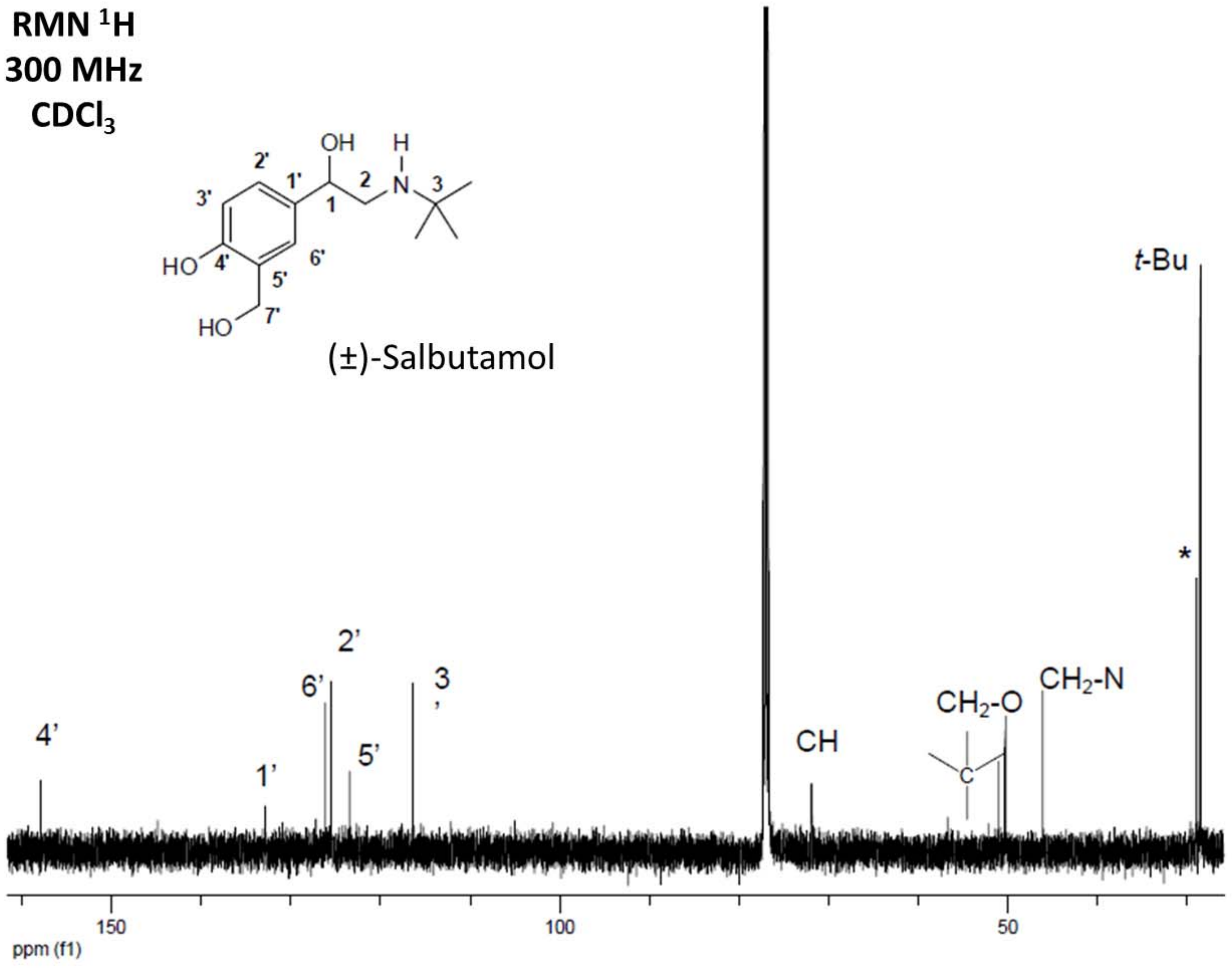

Supplement: Supplementary file 1 [file molecules-17-13864-s001.pdf]
